# Dynamic microtubule association of Doublecortin X (DCX) is regulated by its C-terminus

**DOI:** 10.1038/s41598-017-05340-x

**Published:** 2017-07-12

**Authors:** Maryam Moslehi, Dominic C. H. Ng, Marie A. Bogoyevitch

**Affiliations:** 1 Cell Signalling Laboratories, Department of Biochemistry and Molecular Biology, University of Melbourne, Parkville, Victoria 3010 Australia; 2 Bio21 Institute, Department of Biochemistry and Molecular Biology, University of Melbourne, Parkville, Victoria 3010 Australia; 3 School of Biomedical Sciences, University of Queensland, St Lucia, Queensland, 4072 Australia

## Abstract

Doublecortin X (DCX), known to be essential for neuronal migration and cortical layering in the developing brain, is a 40 kDa microtubule (MT)-associated protein. DCX directly interacts with MTs via its two structured doublecortin (DC) domains, but the dynamics of this association and the possible regulatory roles played by the flanking unstructured regions remain poorly defined. Here, we employ quantitative fluorescence recovery after photobleaching (FRAP) protocols in living cells to reveal that DCX shows remarkably rapid and complete exchange within the MT network but that the removal of the C-terminal region significantly slows this exchange. We further probed how MT organization or external stimuli could additionally modulate DCX exchange dynamics. MT depolymerisation (nocodazole treatment) or stabilization (taxol treatment) further enhanced DCX exchange rates, however the exchange rates for the C-terminal truncated DCX protein were resistant to the impact of taxol-induced stabilization. Furthermore, in response to a hyperosmotic stress stimulus, DCX exchange dynamics were slowed, and again the C-terminal truncated DCX protein was resistant to the stimulus. Thus, the DCX dynamically associates with MTs in living cells and its C-terminal region plays important roles in the MT-DCX association.

## Introduction

Microtubules (MTs), the cytoskeletal polymers of tubulin, are critical contributors to cell mechanics, protein trafficking, signaling events and cell migration^1^. In the brain, the MT cytoskeleton is of particular significance as it shapes the fine structure of neuronal processes and its modulation is critical for neuronal migration^2^. Doublecortin X (DCX) is a developmentally critical MT-associated protein that regulates neuronal MT organization. Pathogenic mutations in DCX have been documented in individuals presenting with brain developmental defects of lissencephaly and subcortical band heterotopia that arise from defects in cortical neuronal layering during embryonic development^3–7^.

Structurally, the DCX protein consists of two homologous doublecortin (DC) domains, DC1 (the N-terminal DC domain) and DC2 (the C-terminal DC domain), linked in tandem via a flexible unstructured region (linker) and additionally flanked by a likely unstructured N-terminal and serine/proline-rich C-terminal sequences^8, 9^. The structured DC domains mediate DCX interaction with MTs, specifically binding directly to the corners of four neighboring tubulin dimers^10^. Indeed, multiple pathogenic mutations have been mapped to these structured DC domains thus emphasizing their importance in the normal functions of DCX as a neuronal MT-associated protein^8, 10^.

Although extensive studies have previously focused on defining the features of DCX-MT interaction interface and the subsequent impact of DCX on MT organization, the dynamics of DCX association with MTs in living cells remain largely unexplored. In this study, we have exploited quantitative fluorescence recovery after photobleaching (FRAP) protocols to reveal unanticipated rapid dynamics in the association of GFP-labelled DCX with MTs in living cells. In addressing the regulatory potential of the DCX unstructured C-terminal sequence, we observed an increased association with MTs, as well as different MT bundling patterns, following the expression of a truncated version of DCX lacking its unstructured C-terminal sequence. Furthermore, dynamics were altered when MTs were altered by the addition of nocadazole or taxol, or alternatively when cells were subjected to hyperosmotic stress. Taken together, our findings emphasize a strikingly dynamic association of DCX with MTs in addition to a regulatory role played by the DCX unstructured C-terminus.

## Results and Discussion

### Doublecortin X (DCX) association with MTs is highly dynamic

The interaction of DCX with MTs has been well defined by previous structural studies of purified proteins *in vitro*, including high resolution cryo-electron microscopy^9, 11^. Whilst those studies reveal the positioning of DCX on the MT lattice, highlighting DCX binding to the corner of four neighboring tubulin dimers^10^, they present a static view of this interaction.

To address the dynamics of the association of DCX with MTs in living cells, we performed fluorescence recovery after photobleaching (FRAP) analyses. For our studies, our choice was the COS-1 cell line because analyses in these cells would not be confounded by endogenous DCX and studies of cytoskeleton organisation and regulation are facilitated by the large, well-spread cytoplasm of these cultured cells^12^. In FRAP protocols, the rate of fluorescence recovery in a defined photobleached zone indicates how fast neighbouring fluorescent molecules arrive to fill the photobleached area. Furthermore, the extent of fluorescence recovery reflects the exchangeable pool of fluorescent molecules, with full recovery indicative of a complete exchange but lower, fractional recoveries indicative of less mobile populations of these fluorescent molecules. FRAP has been instrumental in defining transport kinetics between and within specific intracellular organelles^13–15^ as well revealing the binding dynamics of the non-membrane bound proteins including MTs and the interactions of MTs with associated proteins^16, 17^. In particular, the binding features of MT-associated proteins such as tau and stathmin, have been previously analysed by FRAP protocols^17^.

**Figure 1:**
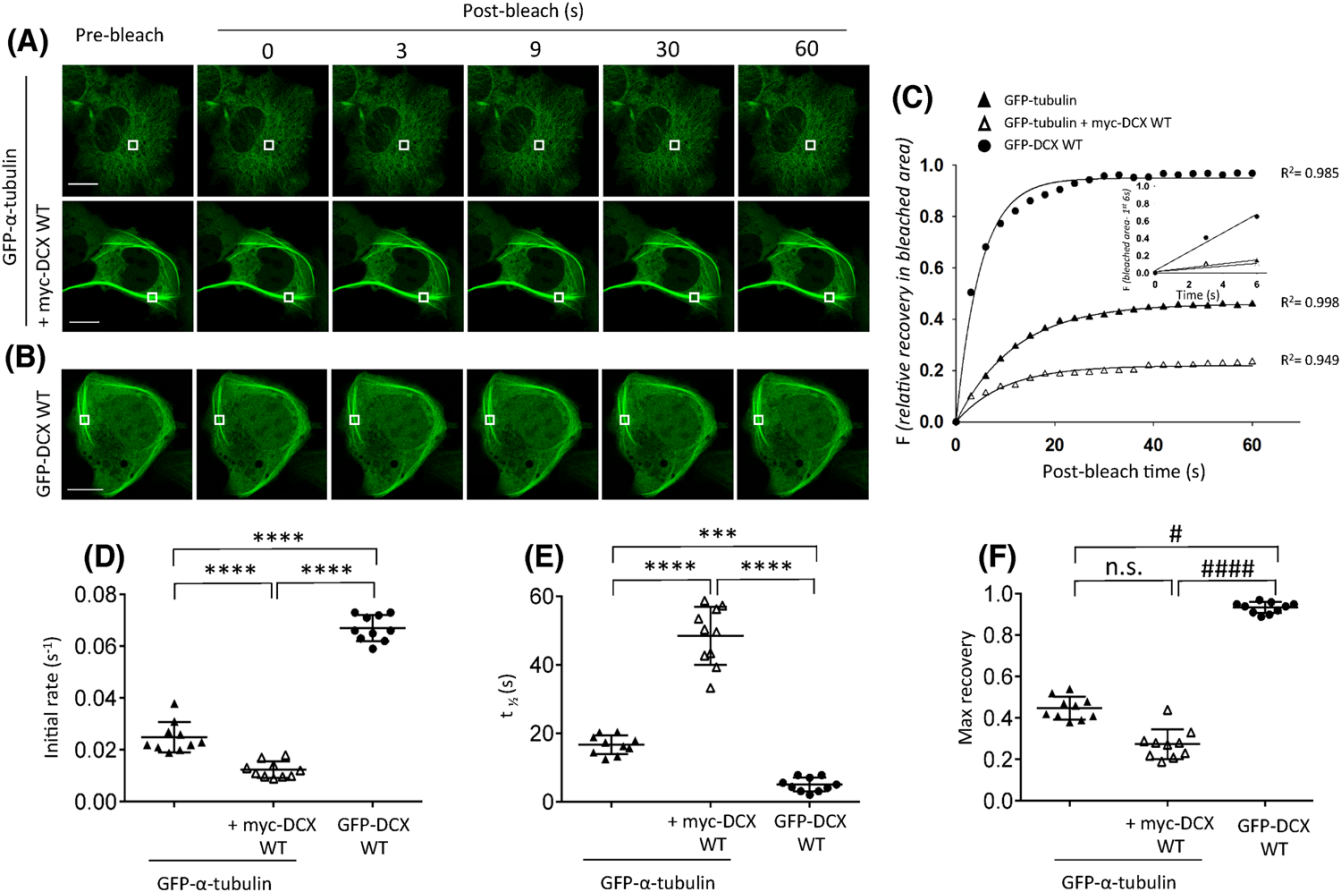
Quantitative FRAP analysis reveals a dynamic association of Doublecortin X (DCX) with stable MT bundles. COS-1 cells were transfected, as indicated, to express GFP-tagged α-tubulin only (upper panels in A), to co-express GFP-α-tubulin together with myc-DCX full-length (wild-type, WT) (lower panels in A) or to express GFP-DCX WT only (**B**). (**A** and **B**) Representative images are shown for quantitative assessments of tubulin or DCX exchange dynamics. A small ROI (indicated by the white rectangle in each cell image) was photobleached and the fluorescence recovery was subsequently monitored post-bleach at 3 s intervals for 60 s for GFP-α-﻿tubulin (**A**) or GFP-DCX WT (**B**). (**C**) The quantitative recovery of fluorescence in each bleach area was determined; regression values (R^2^) of the respective curve fits are indicated. The inset highlights the initial recovery of fluorescence in the photobleached area for first three time-points (0–6 s of post-bleach) with the line of best fit used for the calculation of the initial recovery rate. (**D**–**F**) Pooled quantitative parameters derived from the FRAP data. The recoveries of fluorescence for GFP-α-﻿tubulin, GFP-α-﻿tubulin + myc-DCX WT and GFP-DCX WT over time were analysed. Results are the mean ± SEM for (**D**) the initial rate of recovery of fluorescence for GFP-tubulin or GFP-tubulin + myc-DCX WT and GFP-DCX WT in the bleached area (estimated over the initial 6 s post-bleach as per panel C inset), (**E**) the time to reach half-maximal recovery of fluorescence (t_1/2_) (estimated as per the curve fit in panel C) and (**F**) the fluorescence fractional maximum recovery in the bleached area (where 1.0 represents full-recovery). Error bars represent the standard error of the means and asterisks and hash symbols indicate values calculated as statistically significantly different for the indicated comparisons for parametric and non-parametric analysis, respectively (***p ≤ 0.001, ****p ≤ 0.0001 and ^#^ *p* ≤ 0.05, ^####^ *p* ≤ 0.0001 n = 10 cells in each of the three independent experiments. n.s. = not significant). Scale bars represent 10 µm.

As a control for our FRAP analyses, we initially monitored the recovery of GFP-α-tubulin fluorescence when expressed alone or when co-expressed with wild-type (WT) myc-DCX (Fig. 1A, upper and lower panels, respectively). The progress of fluorescence recovery is further illustrated in the supplementary movie (Supplementary Movie S1A) and the zoom of the photobleached area fluorescence recovery (Supplementary Fig. S1A). We observed a slow recovery of GFP-α-tubulin (Fig. 1C, filled triangle) and of GFP-α-tubulin in the presence of myc-DCX WT (Fig. 1C, open triangle). We extended these observations by quantitative analysis of fluorescence recovery with exponential curve fitting to reveal that the presence of myc-DCX WT further slows both the recovery initial rate (Fig. 1D) and the time to achieve the half-maximal recovery (t_½_) for GFP-α-tubulin (Fig. 1E). Moreover, the presence of myc-DCX WT marginally lowered the maximum recovery of GFP-α-tubulin in the previously bleached area (Fig. 1F), indicative of the actions of DCX to increase the immobilised pool of tubulin within these MT structures. These findings are consistent with the observed increased dynamic exchange of GFP-α-tubulin when MTs were depolymerized by nocodazole or the slowed exchange for MTs bundled by taxol (Supplementary Fig. S2). These analyses reiterate the slow exchange of tubulin within MT structures that can be slowed by MT bundling by either chemical means (taxol) or by the expression of DCX.

We then extended our FRAP analyses to evaluate the dynamics of association of DCX with the MT network by assessing the fluorescence recovery of full-length GFP-tagged DCX. We observed a near-complete recovery of GFP-DCX WT associated with MTs within 20s (Fig. 1B and C filled circle, Supplementary Movie S1B) and the zoom of the photobleached area fluorescence recovery (Supplementary Fig. S1B). Quantitative analysis revealed that the initial rate of recovery for GFP-DCX WT was significantly faster than that recorded for all our assessments of GFP-α-tubulin (Fig. 1C inset, and Fig. 1D) with the time to reach half-maximal recovery (t_½_) being significantly faster than that observed for GFP-α-tubulin either in the presence or in the absence of myc-DCX WT (Fig. 1E). Furthermore, the maximum recovery of GFP-DCX WT in the previously bleached area was significantly higher than observed for tubulin (Fig. 1F), indicative of the near complete and rapid exchange of DCX on these MT structures. Similar results were obtained regardless of the choice of MT bundle thickness in GFP-DCX-WT expressing cells, with calculated values for initial rate of recovery, t_½_, and maximum recovery showing not statistically significant difference when exchange for thick or thin MT bundles were compared (Supplementary Fig. S3; thick MT bundles defined as >1 μm width, thin MT bundles defined as <1 μm width). Furthermore, dynamic exchange of GFP-DCX-WT was observed in the SH-SY5Y neural cell line, extending the relevance of these findings to a neuronal context (Supplementary Fig. S4). Thus, although DCX is known to bind, stabilise and nucleate MTs^18^, our quantitative FRAP analyses reveal that the MT-DCX interaction is one that shows rapid and dynamic exchange of DCX in living cells. This dynamic association with MTs has been largely unappreciated for DCX, but is consistent with recent qualitative observations of rapidly fluctuating speckled patterns of DCX labelling along MTs as assessed in time-lapse recordings in living cells^19^.

### DCX C-terminal deletion increases its MT association and alters MT distribution

**Figure 2:**
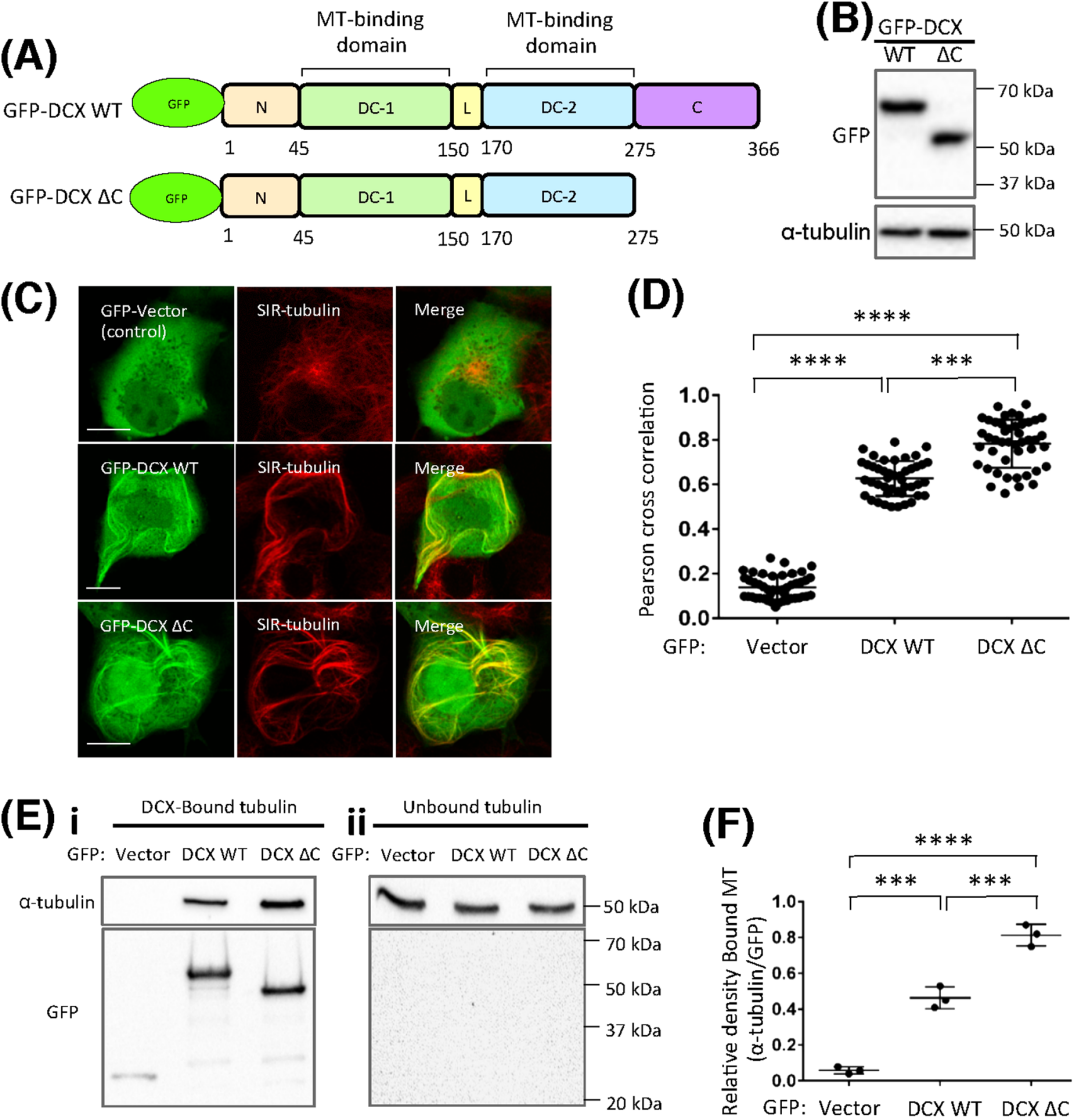
DCX C-terminal deletion increases DCX association with MTs and induces different MT bundling patterns. (**A**) GFP-tagged DCX full-length (wild-type, WT) and a DCX C-terminal truncation mutant lacking residues 276–366 (DCX ΔC) were created to test the impact of the DCX C-terminus on the association of DCX with MTs. (**B**) Following transfection into COS-1 cells, equivalent expression of the GFP-DCX constructs was detected by immunoblotting for GFP (upper panel), with equivalent protein loading detected by immunoblotting for total α-tubulin (lower panel). Blots for both GFP and α-tubulin were cropped. (**C**) GFP-DCX constructs were visualised as green by confocal laser scanning microscopy (left panels), and the impact on endogenous tubulin organisation was evaluated by staining with the live imaging dye SIR-tubulin (red, middle panels); merge images indicate the areas of colocalization (yellow) of GFP and SIR-tubulin. Scale bars represent 10 µm. (**D**) The impact on endogenous tubulin organisation was evaluated by the Pearson correlation coefficient. (**E**) Cells expressing GFP (control vector), GFP-DCX WT and GFP-DCX ΔC were incubated with GFP-trap beads overnight. The GFP-trap beads bound to GFP-tagged constructs were separated from the supernatant by centrifugation. (i) The pellet fraction contains GFP-tagged constructs bound to tubulin (DCX-bound tubulin) and (ii) the supernatant fraction contains soluble tubulin (unbound tubulin). Immunoblotting was performed to detect endogenous α-tubulin (upper panel) or GFP (lower panel). Blots for α-tubulin were cropped. (**F**) Co-precipitation of MTs with GFP-DCX proteins was estimated from quantitating the tubulin/GFP ratio in each pellet fraction containing DCX-bound tubulin. Error bars represent the standard error of the means and asterisks indicate values calculated to be statistically significantly different (**p ≤ 0.01, ***p ≤ 0.001, ****p ≤ 0.0001 n = three independent experiments).

The two DC domains of DCX, DC1 and DC2, show high sequence similarity^8, 11^. Multiple studies indicate that the DC domains are critical for the direct interaction of DCX with MTs^9^. Whilst DC1 and DC2 are separated by a 20 amino acid unstructured linker (L) sequence, two additional predicted unstructured regions form the DCX N-terminus (N) and C-terminus (C)^8^ (Fig. 2A, upper). Because numerous serine/threonine kinases that impact on MT organization are known to target the DCX C-terminal sequence^20–25^, we proposed that the DCX C-terminus contributes to the regulation of DCX-MT interaction dynamics. To evaluate this possibility, we created GFP-DCX ΔC (i.e. GFP-tagged DCX residues 1–275, thus lacking DCX residues 276–366), a C-terminally truncated construct lacking the entire region flanking DC2 (Fig. 2A, lower panel). Both GFP-DCX WT and GFP-DCX ΔC were expressed at comparable levels at their expected sizes (Fig. 2B). In live cell imaging using the dye SIR-tubulin to visualise the MT network, we observed the close co-association of both GFP-DCX WT and GFP-DCX ΔC with tubulin (Fig. 2C). These results, confirmed by quantitative analysis using Pearson cross correlation analysis, further revealed a statistically significantly higher correlation for GFP-DCX ΔC (Fig. 2D). Similar results were obtained for C-terminal GFP fusions of the DCX proteins, DCX WT-GFP and DCX ΔC-GFP (Supplementary Fig. S5A–D). We used a skewness value calculation for N-terminal and C-terminal GFP fusions of the DCX protein to assess their organization within the cytosol. This calculation of the asymmetrical fluorescence distribution also indicated a significant impact by the loss of the DCX C-terminus (Supplementary Fig. S5E and F, respectively) so that the loss of the C-terminus results in a decreased calculated skewness value, i.e. a more symmetrical distribution (wherein skewness calculated as >1 represents highly asymmetrical and skewness 0 = symmetrical).

To assess the interaction of these DCX proteins with MTs, we immunoprecipitated GFP-tagged constructs using “GFP-trap” beads and detected the associated tubulin by immunoblotting the precipitated pellet fraction (Fig. 2E, left panel). Complete capture of GFP-DCX WT and GFP-DCX ΔC by the incubation with GFP-trap beads was confirmed by the absence of these proteins in the supernatant fractions (Fig. 2E, right panel). For comparable levels of immunoprecipitated GFP-DCX proteins, we observed higher tubulin levels associated with GFP-DCX ΔC, a result confirmed by quantitative analysis over 3 independent experiments (Fig. 2F). These results indicate an increased association of DCX ΔC with the tubulin of the MT network, consistent with the higher association with MTs (Fig. 2C,D and Supplementary Fig. S2C,D), observations that could be directly explored in subsequent experiments using live cell imaging and quantitative FRAP protocols. Importantly, these results point to a critical contribution by the DCX C-terminus in the regulation of the DCX-MT interaction.

### DCX C-terminal deletion slows DCX dynamic association with MTs

DCX interactions with MTs have been recently observed to be highly sensitive to MT curvature (i.e. straight versus curved MT structures) with increased DCX binding to the straight, GDP-MT lattice conformation but dissociation from MTs with increasing local curvature^19^. To address how the DCX C-terminus could contribute to interactions with MTs, we extended our quantitative FRAP studies to examine the behaviour of both GFP-DCX WT and GFP-DCX ΔC under conditions of MT depolymerisation (i.e., +Nocodazole) or stabilization (i.e., +Taxol)^19, 26–28^.

**Figure 3:**
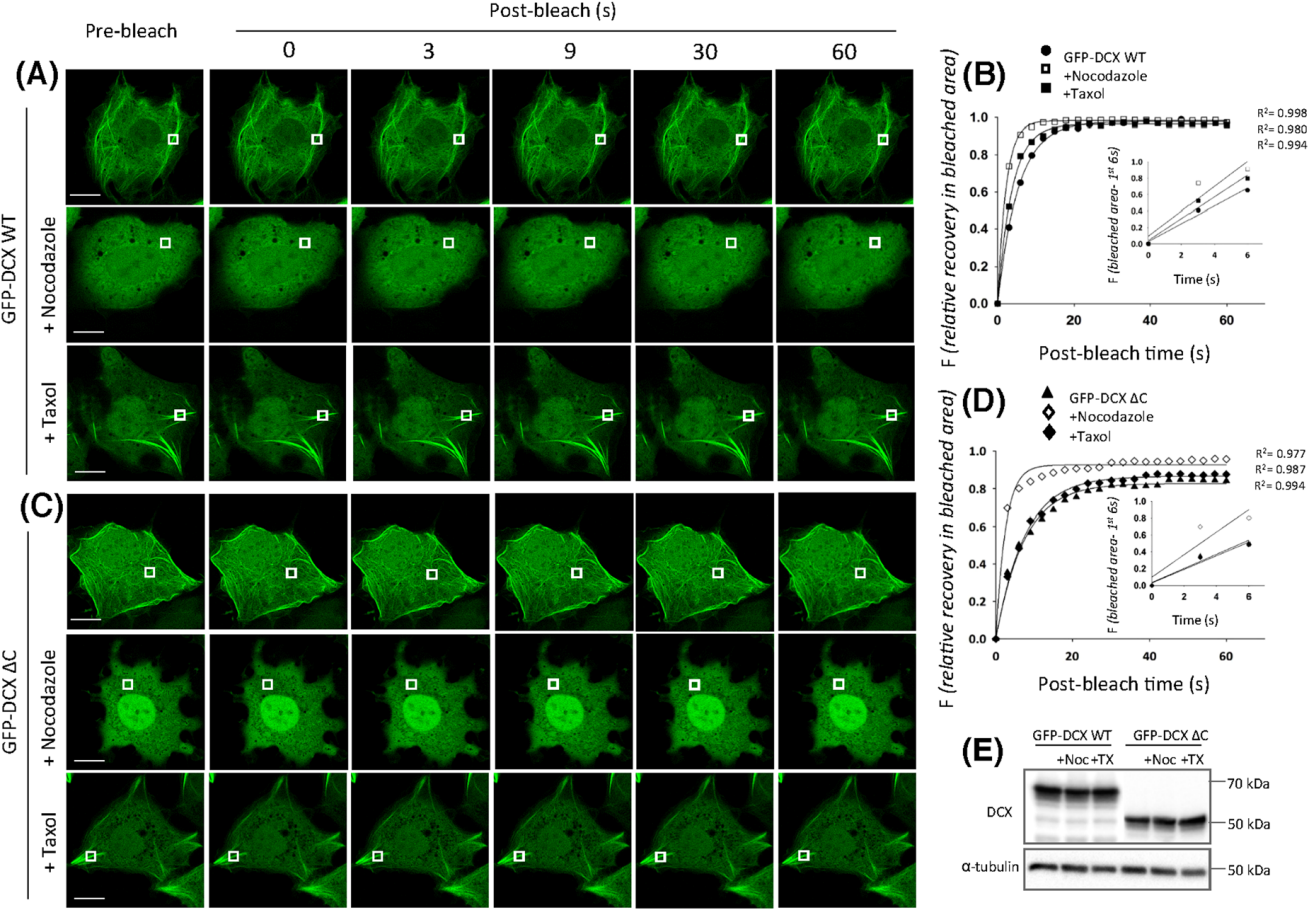
FRAP analysis reveals that GFP-DCX dynamics can be influenced by agents that alter MT stability. COS-1 cells were transfected to express (**A**) GFP-DCX WT or (**C**) GFP-DCX ΔC. Cells were exposed to nocodazole (20 µM, 2 h) to depolymerise MTs or to taxol (10 µM, 1 h) to stabilize MT polymers. A small ROI (indicated by white rectangle in each cell image) was photobleached in (**A**) and (**C**) and the fluorescence recovery was subsequently monitored post-bleach at 3 s intervals for 60 s. Plots of the recovery of fluorescence in the small area of bleach for (**B**) DCX WT and (**D**) DCX ΔC constructs before and after nocodazole or taxol treatments are shown. Regression values for the accuracy of each curve fit are indicated. The insets represent the initial recovery of fluorescence in the photobleached area for first three time-points (0–6 s of post-bleach) with the line of best fit used for the calculation of the initial recovery rates. (**E**) Equivalent expression of the GFP-DCX constructs was detected in the presence and absence of nocodazole or taxol by immunoblotting for DCX (upper panel), with equivalent protein loading detected by immunoblotting for total α-tubulin (lower panel). Scale bars represent 10 µm. See Fig. 4 for quantitative pooled data.

As per our initial FRAP protocol observations (Fig. 1), we observed the rapid and near complete fluorescence recovery for GFP-DCX WT indicative of its high mobility and association with MTs (Fig. 3A upper panels, Fig. 3B filled circles). Treatment with nocodazole (20 µM, 2 h) effectively depolymerised the bundled MT network thus allowing the assessment of GFP-DCX WT in the absence of the complex MT structures and revealing accelerated fluorescence recovery into the bleached areas under these conditions (Fig. 3A middle panels, Fig. 3B open squares). Treatment with taxol (10 µM, 1 h) to stabilize MTs^19, 28^ similarly accelerated GFP-DCX WT fluorescence recovery (Fig. 3A lower panels, Fig. 3B closed squares). The progress of fluorescence recovery is further illustrated in the supplementary movies (Supplementary Movie S2A) and the zoom of the photobleached area fluorescence recovery (Supplementary Fig. S1C).

**Figure 4:**
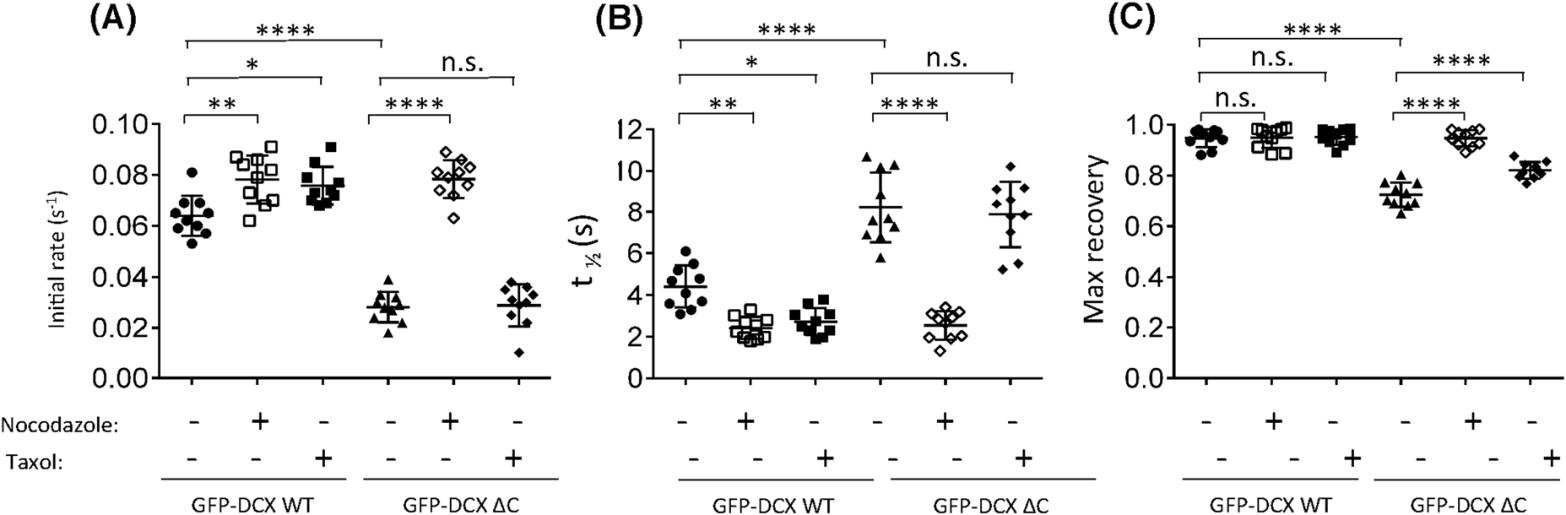
DCX C-terminal deletion slows dynamic association with MTs and decreases sensitivity to taxol-induced MT changes. (**A**–**C**) The recovery of the ROI fluorescence for GFP-DCX WT and GFP-DCX ΔC under basal, nocodazole or taxol treatments. Results are for the mean ± SEM for (**A**) the initial rate of recovery of fluorescence for GFP-DCX WT or GFP-DCX ΔC in the photobleached area (estimated over the initial 6 s post-bleach), (**B**) the time to reach half-maximal recovery of fluorescence (t_1/2_), and (**C**) the fluorescence maximum recovery for GFP-DCX WT or GFP-DCX ΔC in the bleached area. Error bars represent the standard error of the means and asterisks indicate values calculated to be statistically significantly different (*p ≤ 0.05, **p ≤ 0.01, ****p ≤ 0.0001 n = 10 cells in each of three independent experiments. n.s. = not significant).

Quantitative analysis for GFP-DCX WT in the presence of nocodazole or taxol confirmed significantly faster initial rates of recovery (Fig. 4A) and faster times for half-maximal recovery (Fig. 4B), without any significant effect on the maximal recovery (Fig. 4C). Thus, our results suggest that the accessibility of full length DCX to the MT lattice can be enhanced in the presence of taxol, so that recoveries are comparable to those in absence of MT structures in the presence of nocodazole. This differs from previous observations of rapid dissociation of DCX from straight MTs in the presence of the taxol relative paclitaxel^19^ but emphasizes that measurements of binding association are also critical in determining DCX interaction dynamics with MTs. Interestingly, the MT-associated protein tau has also been shown to recognise paclitaxel-induced MT changes, however this sensitivity to paclitaxel has not been observed in other members of the MT-associated protein family^26, 29^. Thus, further studies are needed to address the mechanisms underlying the sensing of MT changes by both Tau and DCX.

To address the possible regulatory role for the DCX C-terminus in dictating the DCX-MT association, we extended our analysis to include a FRAP protocol for GFP-DCX ΔC (Fig. 3C upper panels, Fig. 3D filled triangles). Immunoblot analysis confirmed equivalent expression of the GFP-DCX constructs was detected under these treatment conditions (Fig. 3E). The progress of fluorescence recovery is further illustrated in the supplementary movies (Supplementary Movie S2B) and the zoom of the photobleached area fluorescence recovery (Supplementary Fig. S1D). Quantitative analysis for GFP-DCX ΔC showed its recovery was slower than the recovery of GFP-DCX WT (Fig. 4A), with longer times for half-maximal recovery (Fig. 4B), but with compromised maximal recovery (Fig. 4C). As noted for GFP-DCX-WT, the calculated values for initial rate of recovery, t_½_, and maximum recovery in the presence of GFP-DCX ΔC were not statistically significant difference when exchange for thick or thin MT bundles were compared (Supplementary Fig. S6, thick MT bundles defined as >1 μm width, thin MT bundles defined as <1 μm width). For all parameters, results for GFP-DCX WT and GFP-DCX ΔC were indistinguishable in the presence of nocodazole, consistent with the differences under control conditions reflecting intrinsic properties of these proteins rather than additional intracellular factors. However, we also noted that the initial rates of recovery and the times for half-maximal recovery for GFP-DCX ΔC in the presence of taxol or under control conditions were not significantly different (Fig. 4A,B) albeit that maximal recovery was slightly but significantly improved (Fig. 4C). These results reveal that DCX loses its sensitivity to taxol-induced changes in the absence of its C-terminus, emphasizing a need for improved understanding of the mechanisms of action of taxol as a MT stabilizer^28^.

### The DCX C-terminus can mediate influences of environmental stress on DCX dynamic changes in association with MTs

**Figure 5:**
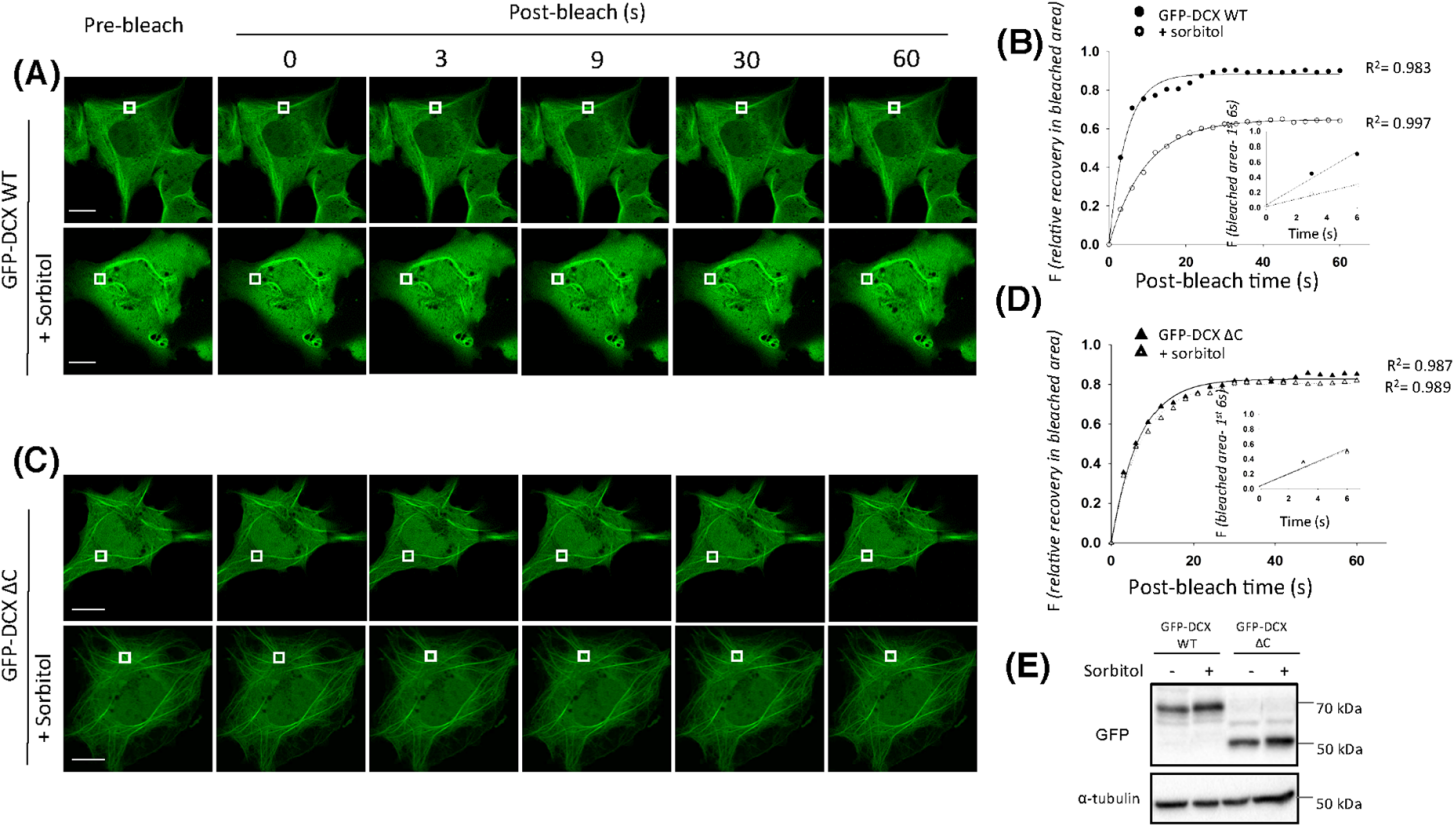
FRAP analysis reveals that hyperosmotic stress conditions impact GFP-DCX dynamics of association with MTs. COS-1 cells were transfected to express (**A**,**B**) GFP-DCX WT or (**C**,**D**) GFP-DCX ΔC. Cells were exposed to sorbitol (0.5 M, 1 h) to induce hyperosmotic stress. A FRAP protocol with a small bleach area and measuring recovery into the bleached area was utilized (**A**–**D**). A small ROI (indicated by the white rectangle in each cell image) was photobleached in (**A**) and (**C**) and the fluorescence recovery subsequently monitored at 3 s intervals for 60 s. Plots of the recovery of fluorescence in small area of bleach in DCX WT (**B**) and DCX ΔC (**D**) constructs in the presence and absence of sorbitol treatments are shown. Regression values for the accuracy of the respective curve fits are shown. Insets represent the recovery of fluorescence in bleach area for first three time-points (0–6 s of post-bleach) with the line of best fit used for the calculation of initial rate. (**E**) Equivalent expression of the GFP-DCX constructs was detected in the presence and absence of sorbitol by immunoblotting for GFP (upper panel), with equivalent protein loading detected by immunoblotting for total α-tubulin (lower panel). Scale bars represent 10 µm. See Fig. 6 for quantitative pooled data.

In the preceding studies, our direct modulation of MT organization influenced DCX-MT association revealing that the DCX C-terminus makes a significant contribution. To address an involvement of the DCX C-terminus in mediating changes following alterations of the cellular environment, we chose to expose cells to environmental stress as initiated by the hyperosmolarity initiated by high concentrations of the non-metabolizable sugar, sorbitol^30^. Thus, we added 0.5 M sorbitol to the cell culture medium, inducing hyperosmotic stress for 1 h prior to further analysis and assessed the dynamics of exchange of GFP-DCX WT and GFP-DCX ΔC proteins in our quantitative FRAP protocols (Fig. 5A and C). For GFP-DCX WT, hyperosmolarity slowed the fluorescence recovery into the bleached area (Fig. 5B) indicating a clear impact on the dynamic association of DCX with the MT network; in contrast we observed no impact of hyperosmolarity on the recovery rate for GFP-DCX ΔC (Fig. 5D). Immunoblot analysis confirmed equivalent expression of the GFP-DCX constructs was detected under these hyperosmotic conditions (Fig. 5E). The progress of fluorescence recovery is further illustrated in the supplementary movies (Supplementary Movie S3A and B) and the zoom of the photobleached area fluorescence recovery (Supplementary Fig. S1E and F).

**Figure 6:**
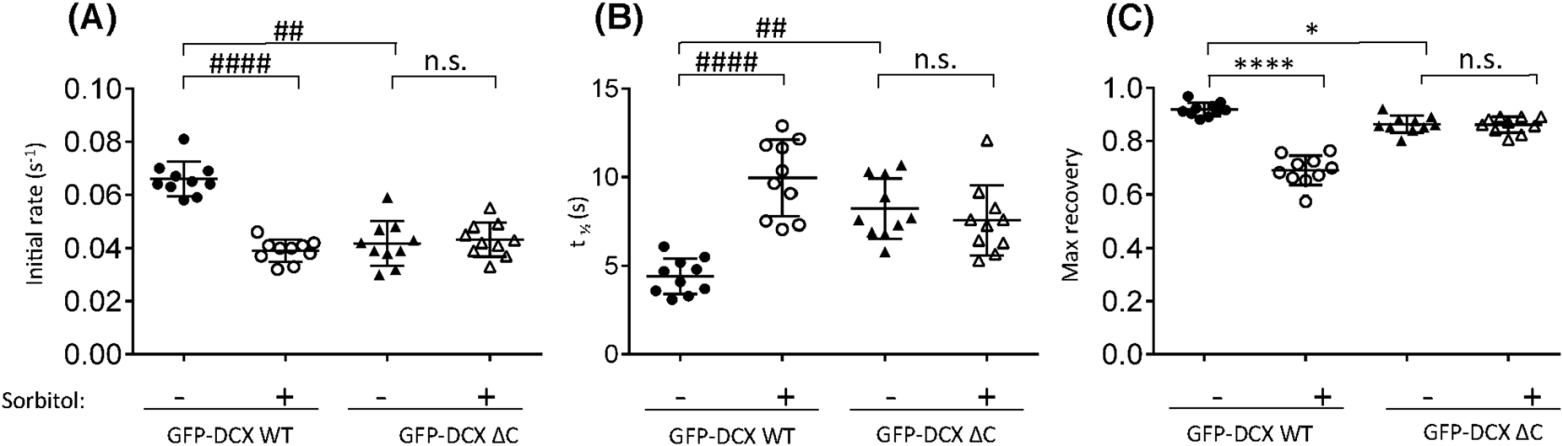
Hyperosmotic stress slows the dynamic association of DCX, but not DCX ΔC, with MTs. (**A**–**C**) The recovery of the ROI fluorescence for GFP-DCX WT and GFP-DCX ΔC under basal or sorbitol treatment conditions. Results are for the mean ± SEM for (**A**) the initial rate of recovery of fluorescence for GFP-DCX WT or GFP-DCX ΔC in the photobleached area (estimated over the initial 6 s post-bleach), (**B**) the time to reach half-maximal recovery of fluorescence (t_1/2_), and (**C**) the fluorescence maximum recovery for GFP-DCX WT or GFP-DCX ΔC in the bleached area. Error bars represent the standard error of the means and asterisks and hash symbols indicate values calculated to statistically significantly different (*p ≤ 0.05, ****p ≤ 0.0001 and ^##^ *p* ≤ 0.01, ^####^ *p* ≤ 0.0001 n = 10 cells in each of three independent experiments. n.s. = not significant).

Quantitative FRAP analysis revealed that sorbitol-induced hyperosmotic stress significantly slowed the rate of fluorescence recovery (Fig. 6A) and the half-time of maximum recovery (Fig. 6B). Moreover, the maximal recovery was decreased for GFP-DCX WT, suggesting that hyperosmotic stress increases the pool of less mobile GFP-DCX WT in association with the MT network (Fig. 6C). However, in the absence of DCX C-terminus, sorbitol-induced hyperosmotic stress did not significantly change the dynamics of association or maximal recovery of GFP-DCX ΔC (Fig. 6A–C) suggesting an important role of the DCX C-terminus in the modulation of DCX dynamics as part of the response to hyperosmotic stress exposure.

**Figure 7:**
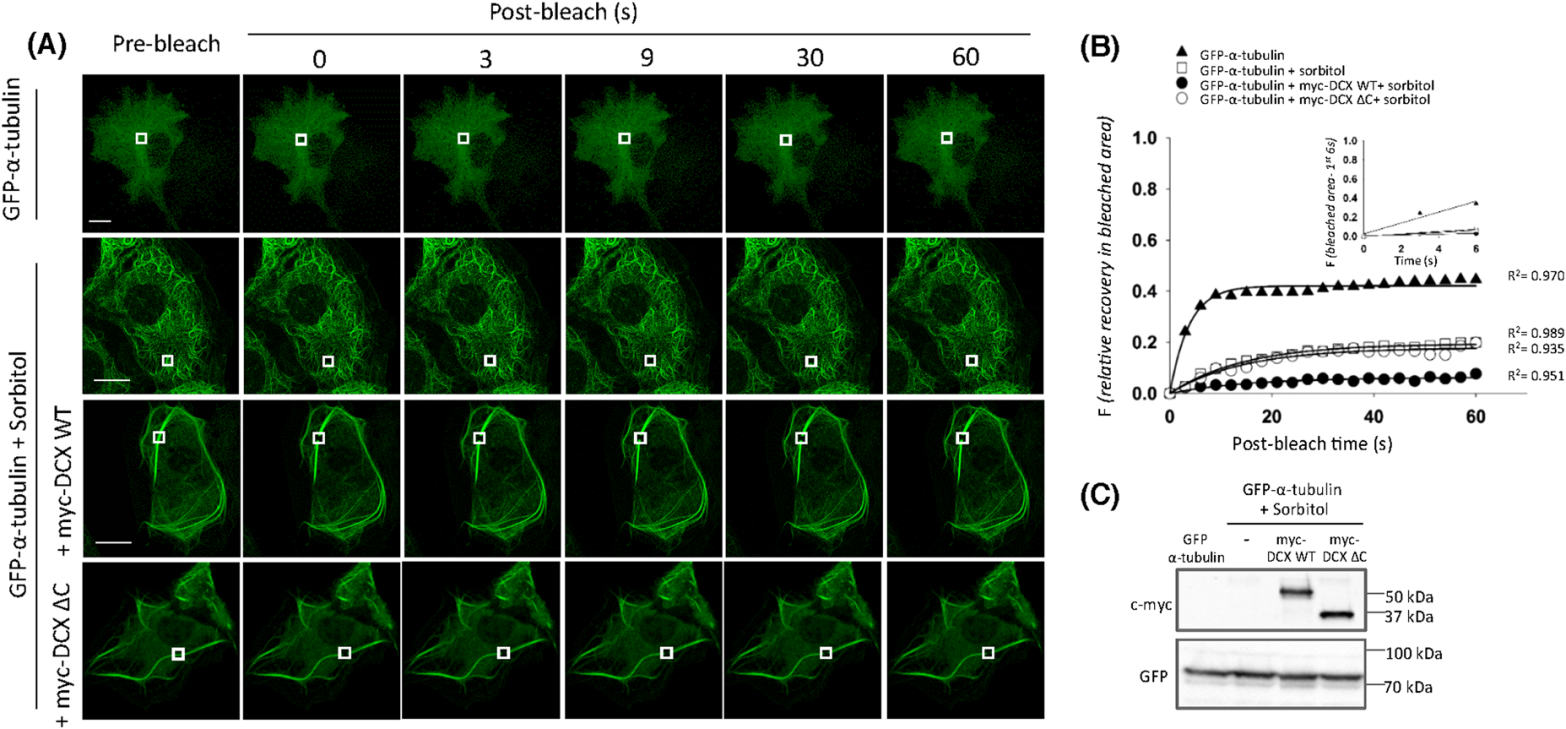
FRAP analysis reveals that hyperosmotic stress conditions affect GFP-α-tubulin exchange dynamics. COS-1 cells were transfected to express (**A**) GFP-α-tubulin, GFP-α-tubulin together with myc-DCX WT or GFP-α-tubulin together with myc-DCX ΔC and then exposed to sorbitol (0.5 M, 1 h) as indicated to induce hyperosmotic stress. A FRAP protocol with a small bleach area and measuring recovery into the bleached area was utilized (**A**,**B**). A small ROI (indicated by the white rectangle in each cell image) was photobleached in (**A**) and the fluorescence recovery subsequently monitored at 3 s intervals for 60 s. (**B**) Plots of the recovery of fluorescence in small area of bleach in GFP-α-tubulin with or without myc-DCX constructs in the presence and absence of sorbitol treatments are shown. Regression values for the accuracy of the respective curve fits are shown. Insets represent the recovery of fluorescence in bleach area for first three time-points (0–6 s of post-bleach) with the line of best fit used for the calculation of initial rate. (**C**) Equivalent expression of the GFP-α-tubulin and myc-DCX constructs was detected in the presence and absence of sorbitol by immunoblotting for c-myc (upper panel) and GFP (lower panel). Scale bars represent 10 µm. See Fig. 8 for quantitative pooled data.

**Figure 8:**
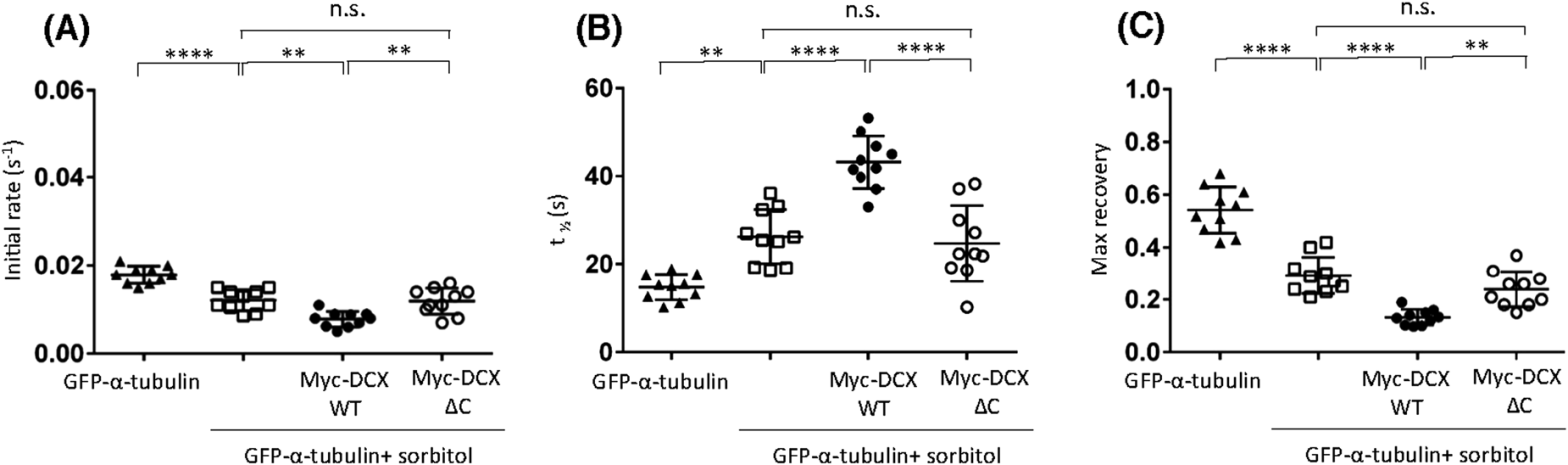
Hyperosmotic stress slowing of tubulin dynamic change is further slowed by DCX WT but not DCX ΔC. (**A**–**C**) The recovery of the ROI fluorescence for GFP-α-tubulin only and together with myc-DCX WT or myc-DCX ΔC under sorbitol treatment conditions. Results are for the mean ± SEM for (**A**) the initial rate of recovery of fluorescence for GFP-α-tubulin only or together with myc-DCX WT or myc-DCX ΔC under sorbitol treatment conditions in the photobleached area (estimated over the initial 6 s post-bleach), (**B**) the time to reach half-maximal recovery of fluorescence (t_1/2_), and (**C**) the fluorescence maximum recovery for GFP-α-tubulin in the bleached area. Error bars represent the standard error of the means and asterisks indicate values calculated to statistically significantly different **p ≤ 0.01, ****p ≤ 0.0001, n = 10 cells in each of three independent experiments. n.s. = not significant).

Lastly, as an impact of hyperosmotic stress on tubulin exchange dynamics may also underlie the altered DCX dynamic exchange we observed, we applied our quantitative FRAP protocols to assess GFP-α-tubulin in the presence of sorbitol and also in the presence of DCX WT and DCX ΔC (Fig. 7A–C). The progress of fluorescence recovery is further illustrated in the supplementary movies (Supplementary Movie S4A and B) and the zoom of the photobleached area fluorescence recovery (Supplementary Fig. S1G and H). We observed that hyperosmolarity slowed the GFP-α-tubulin fluorescence recovery into the bleached area, with further slowing in the presence of DCX WT but not DCX ΔC (Fig. 7B). Immunoblot analysis confirmed equivalent expression of the myc-DCX constructs was detected under these hyperosmotic stress conditions (Fig. 7C). For all parameters, expression of DCX ΔC did not further influence the changes noted under hyperosmotic stress (Fig. 8). These results thus further emphasize the critical requirement for the DCX C-terminus in influencing tubulin exchange.

Taken together, our studies probing DCX-MT interactions highlight a dynamic relationship that is largely unexpected when considering previous cryoEM structural studies^9, 11^. Importantly, rapidly exchanging pools of other MT-associated proteins have been recently revealed, including the very fast dynamics of Tau-MT interactions that are critical in promoting neuronal MT assembly without impeding MT-dependent intracellular transport events^31^. For DCX, we have demonstrated that its interaction with MTs is sensitive to MT stabilization, with the likely unstructured DCX C-terminus significantly contributing. It will now be important to further probe how the C-terminus of DCX can influence the DC domain interactions with MTs, particularly as the N-terminal and C-terminal regions flanking the DC domains of DCX remain unresolved in structural studies so that their positioning relative to the DC domains and the MT surface are unknown. Given that the C-terminus is a target for multiple different serine/threonine kinases including c-Jun N-terminal kinase, Cyclin-dependent kinase-5 and protein kinase A^20–24^, the role of phosphorylation-dependent regulation, either by allosteric modulation of DCX itself or via recruitment of additional DCX-interacting partners, should be a top priority for future exploration.

## Methods

### Mammalian expression plasmids

The coding region of human DCX was amplified (source plasmid = pGEX-4T1-DCX full-length^12^) with appropriate cloning sites, by PCR (i.e. XhoI and HindIII restriction sites for GFP-DCX WT and myc-DCX WT, EcoRI and KpnI restriction sites for DCX WT-GFP). The PCR primer pairs are listed in Supplementary Table S1. pGFP-C3, pGFP-N3 and pXJ40-myc plasmid vectors (Addgene) were used to generate the DCX WT-GFP, GFP-DCX WT and myc-DCX WT constructs, respectively. We also generated GFP-tagged DCX C-terminally truncated constructs lacking the entire region flanking DC2 (i.e. DCX residues 1–275, thus lacking DCX residues 276–366): GFP-DCX ΔC and DCX ΔC-GFP. All constructs were validated by restriction digestion and full sequence analysis.

### Cell culture, transfection and treatments

Cells (monkey fibroblast-like Cos-1 line or the human neuroblast SH-SY5Y line) were maintained (37 °C, 5% CO_2_) in growth media (high-glucose Dulbecco’s modified Eagle’s medium (DMEM) for Cos-1 or DMEM-F12 for SH-SY5Y) containing L-glutamine supplemented with 10% (v/v) fetal bovine serum (FBS), 1 mM penicillin and streptomycin. Transient transfections were performed using Lipofectamine^TM^ 2000 and antibiotic-free Opti-MEM medium according to the manufacturer’s instructions (Invitrogen). Following transfection for 24 h and where indicated, cells were further treated with nocodazole (20 µM, 2 h), taxol (10 µM, 1 h) or sorbitol (0.5 M, 1 h) prior to further analysis. For selected experiments, the neuronal (neuroblast) cell line SH-SY5Y was maintained under similar conditions.

### Cell lysis, immunoblotting and immunoprecipitation

Cell lysates were prepared using RIPA buffer [50 mM Tris-HCl, pH 7.3, 150 mM NaCl, 0.1 mM ethylenediaminetetraacetic acid (EDTA), 1% (v/v) sodium deoxycholate, 1% (v/v) Triton X-100, 0.2% (w/v) NaF and 100 µM Na_3_VO_4_] supplemented with complete protease inhibitor mix (Roche Diagnostic). Cell lysates were incubated on ice (10 min) and cleared by centrifugation (16,000 g, 10 min). Protein concentrations were determined by the Bradford assay (BioRad). Protein samples, diluted with 3x protein sample buffer, were resolved by SDS-PAGE [8% (v/v) polyacrylamide gels] and the separated proteins were transferred onto polyvinylidene difluoride (PVDF) membranes (Amersham Life Science).

Proteins of interest were detected using the following primary antibodies: anti-GFP (Abcam 13970), anti-DCX (ThermoFisher Scientific 48–1200) and anti-α-tubulin (Sigma T6074). After incubation with horseradish peroxidase-linked secondary antibodies (ThermoFisher Scientific), immunoreactive proteins were visualised using an enhanced chemiluminescence detection system (ThermoFisher Scientific) and quantitation carried out using FIJI 2.0 public domain software (National Institutes of Health).

Immunoprecipitation was performed by incubating protein lysates (2 mg) together with GFP-trap beads (ChromoTek) at 4 °C overnight, with continuous end-to-end mixing on a rotating wheel. GFP-trap beads were separated from supernatant by centrifugation (16,000 g, 30 s) and beads were thoroughly washed (3x washing with 0.5 ml lysis buffer) with lysis buffer before precipitated proteins were eluted by the addition of 3x protein sample buffer and boiling (100 °C, 5 min). Precipitated protein and supernatants were resolved by SDS-PAGE and immunoblotted as above.

### Live-cell imaging microscopy and image analysis

COS-1 cells were seeded on eight-well µ-slides (Ibidi) and transiently transfected with plasmids encoding GFP-DCX WT or GFP-DCX ΔC. At 24 h post-transfection, the culture medium was replaced with fresh medium containing a final concentration of 10 nM SiR-tubulin dye (Cytoskeleton Inc.). After additional incubation (12 h) with the SiR-tubulin dye, the culture medium was replaced with pre-warmed Phenol-Red-free DMEM growth medium. Images were captured on a Leica TCS SP5 confocal microscope equipped with an environmental chamber (37 °C, 5% CO_2_) using a 63x 0.9 NA objective (Biological Optical Microscopy Platform, University of Melbourne).

The Pearson correlation coefficient was applied to quantify the degree of colocalisation between fluorophores using FIJI software. A higher Pearson correlation coefficient corresponds to greater colocalisation of MTs with DCX constructs^32^.

To study the distribution of MT bundles, a skewness value analysis was applied using FIJI image analysis software. The skewness moment calculator plugin of FIJI measures the asymmetrical fluorescence distribution; specifically the skewness value (Sk) is calculated as:

1 $${\rm{Sk}}=\frac{3(Mean-Median)}{S{tandard}\,deviation}$$

Thus, the interpretation of the spatial moments by skewness value in x and y corresponds to Sk = 0 indicating a symmetric distribution of the detected fluorescent tag, but Sk < 0 or > 0 indicating asymmetric distribution of the detected fluorescent tag. In our study, a higher skewness (Sk > 0) value corresponds to a more heterogeneous distribution of MTs^33^.

### Fluorescence recovery after photobleaching (FRAP) analysis

Photobleach analyses were performed with cells maintained in Phenol-Red-free growth medium at 37 °C with 5% CO_2_ supplied to the microscope chamber. Images were captured on a Leica TCS SP5 confocal microscope equipped with an environmental chamber (37 °C, 5% CO_2_) using a 63x 0.9 NA objective (Biological Optical Microscopy Platform, University of Melbourne). Ten prebleach images were collected with excitation at 488 nm prior to photobleaching. A small region of interest (ROI) was then photobleached (1 s, 100% laser power). Each ROI encompassed MT structures typical of the treatment condition (e.g. thick MT bundles upon DCX expression). Where indicated, additional comparisons of different MT bundles in individual cells were performed, with results recorded for thick MT bundles (defined as >1 μm width) and thin MT bundles (defined as <1 μm width).

Fluorescence recovery was recorded at 3-s intervals for 60 s. FRAP measurements (n = 10) from three independent experiments were normalized as described previously^27, 34^:

2 $${{\rm{F}}}_{\mathrm{frap}-\mathrm{norm}({\rm{t}})}=({{\rm{F}}}_{\mathrm{whole}-\mathrm{prebleach}}/[{{\rm{F}}}_{{\rm{whole}}({\rm{t}})}-{{\rm{F}}}_{{\rm{bg}}({\rm{t}})}])({{\rm{F}}}_{{\rm{frap}}({\rm{t}})}-{{\rm{F}}}_{{\rm{bg}}({\rm{t}})}/{{\rm{F}}}_{\mathrm{frap}-\mathrm{prebleach}})$$

where F_frap(t)_ is the fluorescence recovery in the photobleached ROI at time *t*, F_whole(t)_ is the whole-cell fluorescence and F_bg(t)_ is the background region fluorescence intensity (outside the cell). F_frap-prebleach_ and F_whole-prebleach_ represent mean prebleach fluorescence intensity of the photobleached ROI and the whole cell, respectively.

Normalised FRAP measurements were plotted against postbleach recovery time and the resulting data fitted with the double exponential equation:

3 $$[{{\rm{F}}}_{({\rm{t}})}={{\rm{A}}}_{1}(1-{\exp }^{-{\rm{\tau }}1.{\rm{t}}})+{{\rm{A}}}_{2}(1-{\exp }^{-{\rm{\tau }}2.{\rm{t}}})]$$

to adjust amplitudes A_1_ and A_2_ and time constants ^τ1^ and ^τ2^.

### Statistical analysis

Statistical analyses of data sets for deviation from normal distribution were carried out with GRAPHPAD PRISM 6 software using the D’Agostino-Pearson omnibus normality test. Data sets with normal distribution were analyzed using unpaired two-tailed student’s *t*-test analyses of differences between the control and test conditions (*p ≤ 0.05, **p ≤ 0.01, ***p ≤ 0.001, ****p ≤ 0.0001) whereas data sets with deviation from normal distribution were analyzed using Mann-Whitney and Kruskal-Wallis non-parametric test (^#^ *p* ≤ 0.05, ^##^ *p* ≤ 0.01, ^###^ *p* ≤ 0.001, ^####^ *p* ≤ 0.0001) as indicated.

## Electronic supplementary material

Supplementary Figures and Table

Key to Supplementary Movies S1-S4

Movie S1A GFP-tubulin

Movie S1A GFP-tubulin+myc-DCX WT

Movie S1B GFP-DCX WT

Movie S2A GFP-DCX WT

Movie S2A GFP-DCX WT+nocodazole

Movie S2A GFP-DCX WT+Taxol

Movie S2B GFP-DCX delta C

Movie S2B GFP-DCX delta C+Nocodazole

Movie S2B GFP-DCX delta C+Taxol

Movie S3A GFP-DCX WT

Movie S3A GFP-DCX WT+Sorbitol

Movie S3B GFP-DCX deltaC

Movie S3B GFP-DCX deltaC+Sorbitol

Movie S4A GFP-alpha-tubulin

Movie S4B GFP-alpha-tubulin+sorbitol

Movie S4B GFP-alpha-tubulin+mycDCX WT+sorbitol

Movie S4B GFP-alpha-tubulin+myc-DCX deltaC+sorbitol
