# Supplementary material for: Dynamic microtubule association of Doublecortin X (DCX) is regulated by its C-terminus
Source: Sci Rep. 2017 Jul 12;7:5245. doi: 10.1038/s41598-017-05340-x (PMC5507856; doi:10.1038/s41598-017-05340-x)
Supplement: Supplementary file 1 — Supplementary Figures and Table [file 41598_2017_5340_MOESM1_ESM.pdf]

Dynamic microtubule association of Doublecortin X (DCX) is regulated by its C-terminus

Maryam Moslehi, Dominic C.H. Ng, and Marie A. Bogoyevitch

Supplementary

Figure S1: Zoom images highlight photobleached area fluorescence recovery.
Figure S2: FRAP analysis reveals that GFP-α–tubulin dynamics can be influenced by agents that alter MT organization
Figure S3: FRAP analysis shows that GFP-DCX WT dynamics are not significantly influenced by MT bundle thickness in individual cells.
Figure S4: FRAP analysis reveals the dynamic association of GFP-DCX WT in a neuronal cell line.
Figure S5: Position of GFP tag on DCX termini does not affect DCX-microtubule association and MT organization.
Figure S6: FRAP analysis shows that GFP-DCX ΔC dynamics are not significantly influenced by MT bundle thickness in individual cells.

Table S1: Primers and Sequences

Figure S1

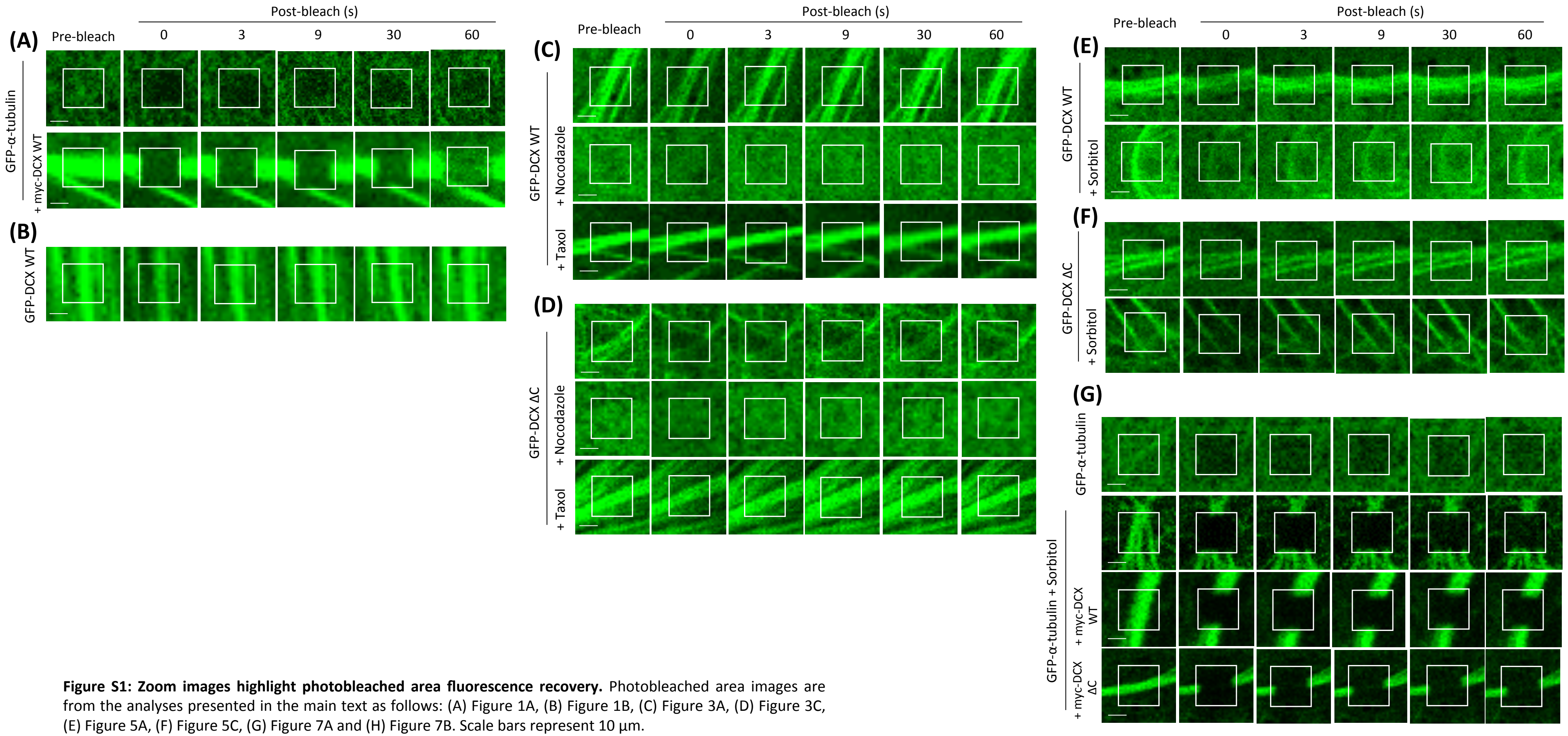

Figure S1: Zoom images highlight photobleached area fluorescence recovery. Photobleached area images are from the analyses presented in the main text as follows: (A) Figure 1A, (B) Figure 1B, (C) Figure 3A, (D) Figure 3C, (E) Figure 5A, (F) Figure 5C, (G) Figure 7A and (H) Figure 7B. Scale bars represent 10 µm.

Figure S2

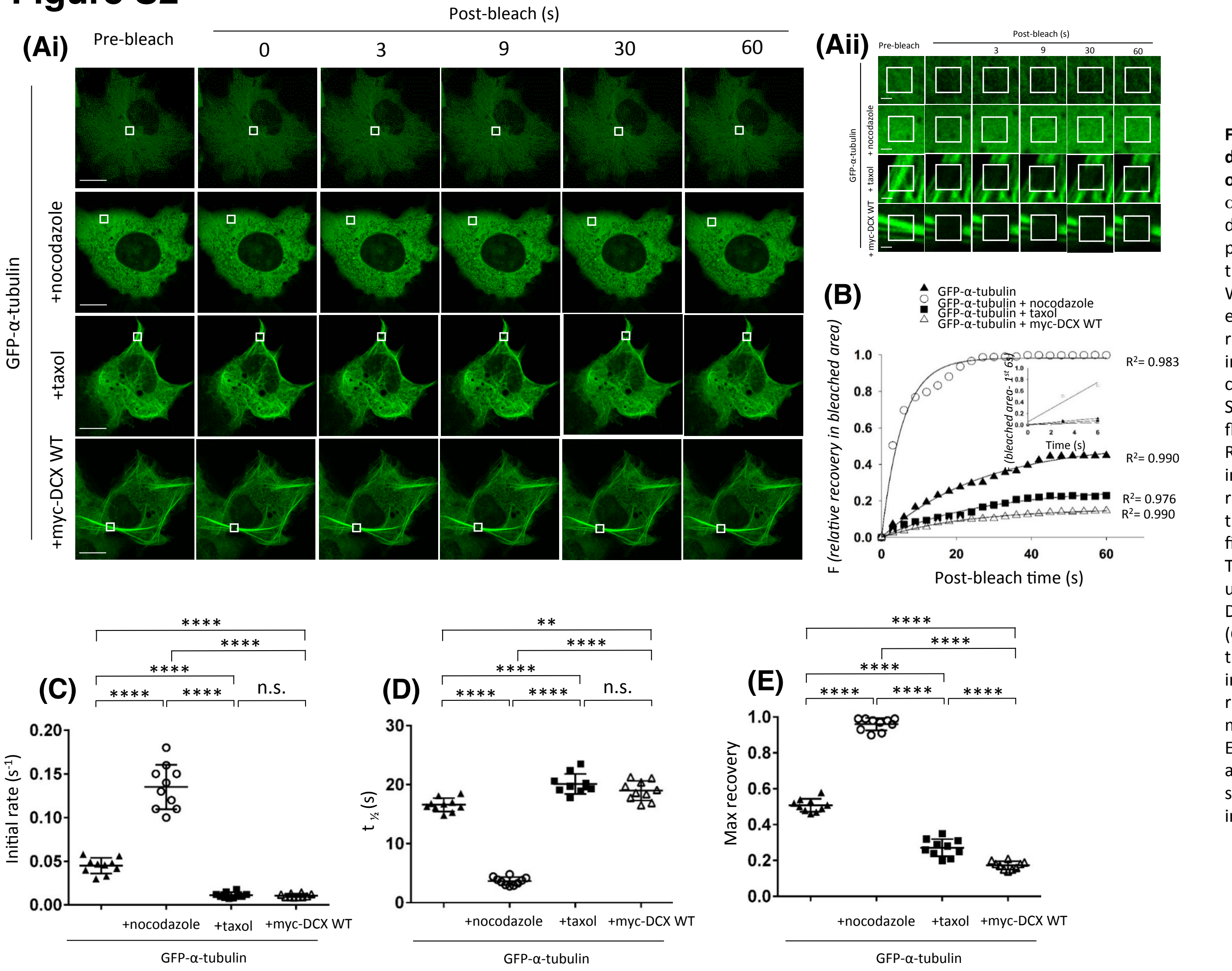

Figure S2: FRAP analysis reveals that GFP-α–tubulin dynamics can be influenced by agents that alter MT organization. COS-1 cells were transfected to express GFP-α-tubulin then exposed to nocodazole (20 μM, 2 h) to depolymerise MTs or to taxol (10 μM, 1 h) to stabilize MT polymers. In parallel experiments, COS-1 cells were transfected to express both GFP-α-tubulin and myc-DCX-WT. (Ai) A small ROI (indicated by the white rectangle in each cell image) was photobleached and the fluorescence recovery was subsequently monitored post-bleach at 3s intervals for 60 s. (Aii) Zoom images highlight the corresponding photobleached area fluorescence recovery. Scale bars represent 10 μm. (B) Plots of the recovery of fluorescence in the small area of bleach are shown. Regression values for the accuracy of each curve fit are indicated. The data in the inset represents the initial recovery of fluorescence in the photobleached area for first three time-points (0-6 s of post-bleach) with the line of best fit used for the calculation of the initial recovery rates. (C-E) The recovery of the ROI fluorescence for GFP-α-tubulin under basal, nocodazole or taxol treatments or with myc-DCX WT co-expression. Results are for the mean ± SEM for (C) the initial rate of recovery of fluorescence for GFP-α-tubulin in the photobleached area (estimated over the initial 6s post-bleach), (D) the time to reach half-maximal recovery of fluorescence ($t_{1/2}$), and (E) the fluorescence maximum recovery for GFP-a-tubulin in the bleached area. Error bars represent the standard error of the means and asterisks indicate values calculated to be statistically significantly different (** $p \leq 0.01$, **** $p \leq 0.0001$ n = 10 cells in 3 independent experiments. n.s.= not significant).

Figure S3

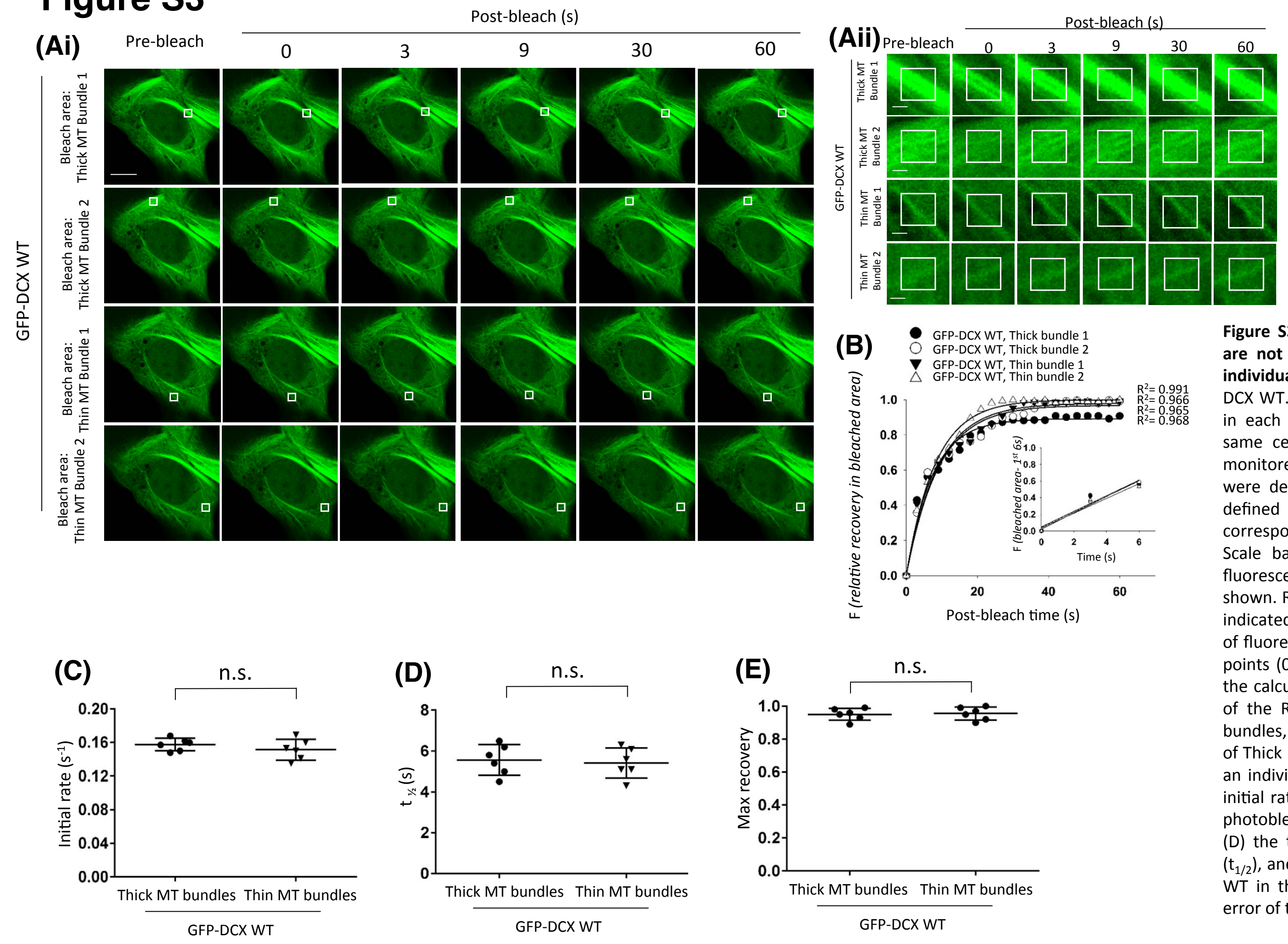

Figure S3: FRAP analysis shows that GFP-DCX WT dynamics are not significantly influenced by MT bundle thickness in individual cells. COS-1 cells were transfected to express GFP-DCX WT. (Ai) Different small ROI (indicated by white rectangle in each cell image) were photobleached sequentially in the same cell and the fluorescence recovery was subsequently monitored post-bleach at 3s intervals for 60 s. Thin MT bundles were defined as < 1 μm width and thick MT bundles were defined as < 1 μm width. (Aii) Zoom images highlight the corresponding photobleached area fluorescence recovery. Scale bars represent 10 μm. (B) Plots of the recovery of fluorescence in the small area of bleach for GFP-DCX WT are shown. Regression values for the accuracy of each curve fit are indicated. The data in the inset represents the initial recovery of fluorescence in the photobleached area for first three time-points (0-6 s of post-bleach) with the line of best fit used for the calculation of the initial recovery rates. (C-E) The recovery of the ROI fluorescence for GFP-DCX-WT for thick and thin bundles, where each data point shown is the average thickness of Thick Bundle 1 and 2, or Thin Bundle 1 and 2, as indicated in an individual cell. Results are for the mean ± SEM for (C) the initial rate of recovery of fluorescence for GFP-DCX WT in the photobleached area (estimated over the initial 6s post-bleach), (D) the time to reach half-maximal recovery of fluorescence ($t_{1/2}$), and (E) the fluorescence maximum recovery for GFP-DCX WT in the bleached area. Error bars represent the standard error of the means; n = 6 cells. (n.s.= not significant).

Figure S4

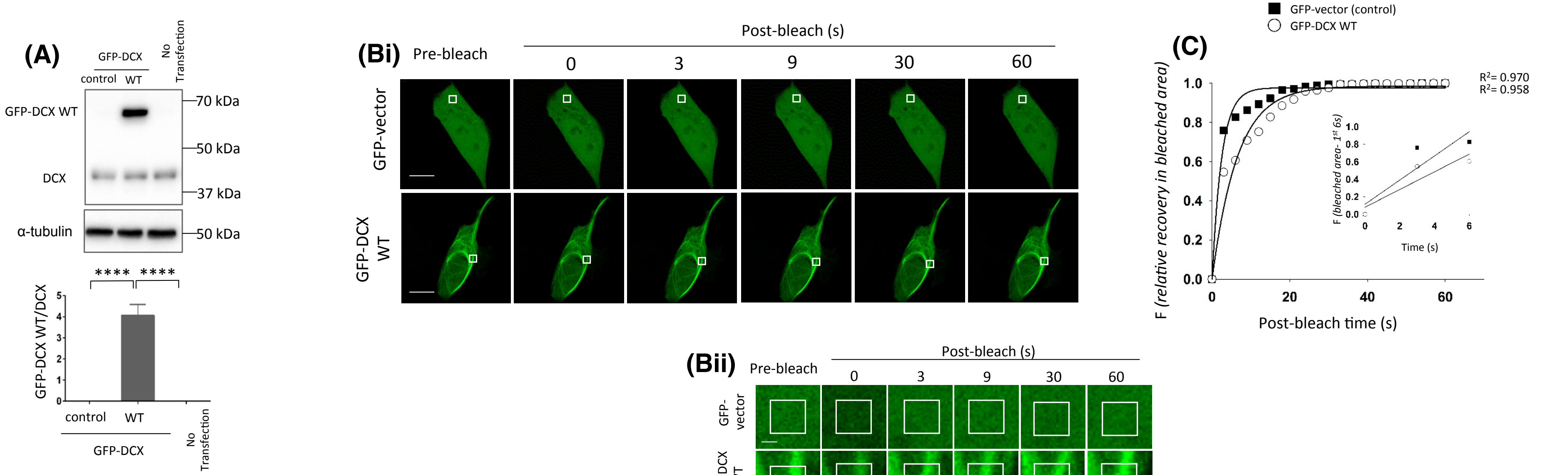

Figure S4: FRAP analysis reveals the dynamic association of GFP-DCX WT in a neuronal cell line. SH-SY5Y cells were transfected to express GFP-DCX WT, the GFP-vector only (control) or left untransfected (no transfection). (A) Immunoblot analysis was performed using anti-DCX antibodies (upper panel) or anti-α-tubulin antibodies (lower panel) as a loading control. (Bi) For cells expressing GFP-DCX-WT or the GFP-vector, a small ROI (indicated by white rectangle in each cell image) was photobleached and the fluorescence recovery was subsequently monitored post-bleach at 3s intervals for 60 s. (Bii) Zoom images highlight the corresponding photobleached area fluorescence recovery. Scale bars represent 10 µm. (C) Plots of the recovery of fluorescence in the small area of bleach for GFP-DCX WT are shown. Regression values for the accuracy of each curve fit are indicated. The data in the insets represent the initial recovery of fluorescence in the photobleached area for first three time-points (0-6 s of post-bleach) with the line of best fit used for the calculation of the initial recovery rates. (D-F) The recovery of the ROI fluorescence for GFP or GFP-DCX-WT. Results are for the mean ± SEM for (D) the initial rate of recovery of fluorescence for GFP or GFP-DCX WT in the photobleached area (estimated over the initial 6s post-bleach), (E) the time to reach half-maximal recovery of fluorescence ($t_{1/2}$), and (F) the fluorescence maximum recovery for GFP or GFP-DCX WT in the bleached area. Error bars represent the standard error of the means and asterisks indicate values calculated to be statistically significantly different (*** *p*≤0.001, *****p*≤0.0001, n = 10 cells in each of three independent experiments. (n.s.= not significant).

Figure S5

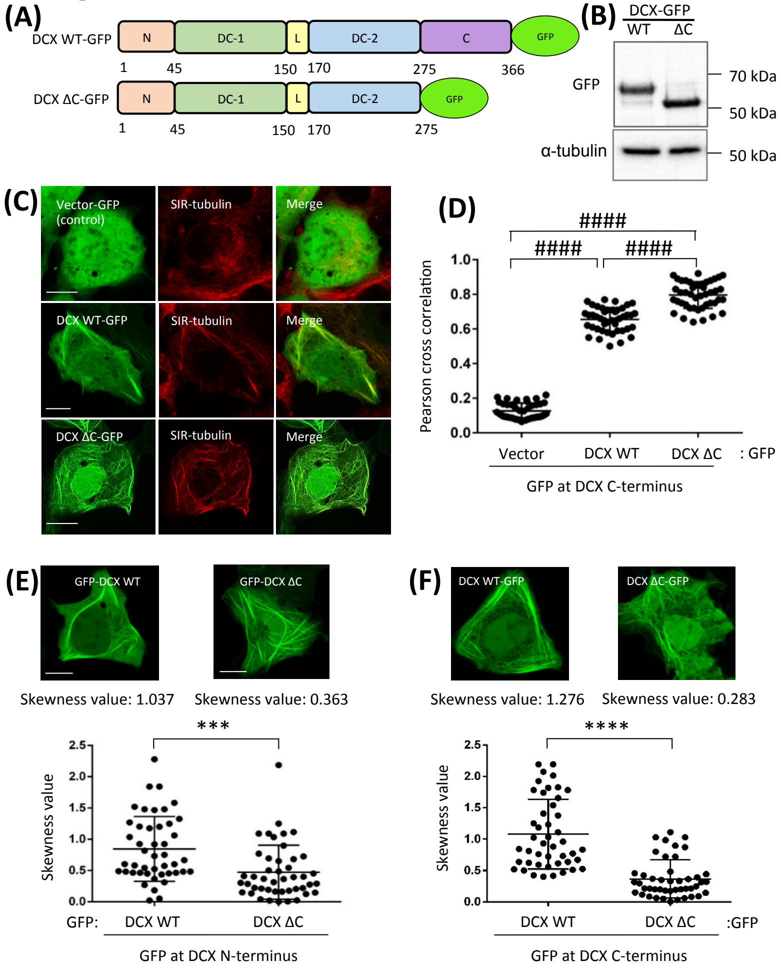

Figure S5: Position of GFP tag on DCX termini does not affect DCX-microtubule association and MT organization. (A) DCX full-length (wild-type, WT) and a C-terminal truncation mutant lacking residues 276-366 (DCX ΔC) with a GFP tag at C-terminus of DCX constructs were created to test the impact of the position of GFP tag on the association of DCX constructs with microtubules. (B) Following transfection into COS-1 cells, the equivalent expression of the DCX-GFP constructs was detected by immunoblotting for GFP (upper panel), with equivalent protein loading detected by immunoblotting for total α-tubulin (lower panel). (C) GFP (vector control) or DCX-GFP constructs were visualised as green by confocal scanning laser microscopy (left panels), and the impact on endogenous tubulin organisation was evaluated by staining as red with the live imaging dye SIR-tubulin (middle panels); merge images indicate the areas of colocalization (yellow) of GFP constructs and SIR-tubulin (right panels). Scale bars represent 10 µm. (D) The colocalization of GFP, DCX WT-GFP or DCX ΔC-GFP with tubulin (SIR-tubulin) was calculated as the Pearson correlation coefficient. Error bars represent the standard error of the means and asterisks indicate values statistically significantly different (#### $p \leq 0.0001$, n= 45 individual cells in three independent experiments). (E) Bundling pattern in both DCX WT and DCX ΔC constructs with GFP tag at N-terminus and (F) at C-terminus were analysed using skewness value ranging between -1 and +1 (-1 for homogeneous and +1 for heterogeneous MT distribution). Images are representatives for each group. Scale bars represent 10 µm. Error bars represent the standard error of the means and asterisks indicate values statistically significantly different (*** $p \leq 0.005$, **** $p \leq 0.0001$ in three independent experiments, n = 45 cells).

Figure S6

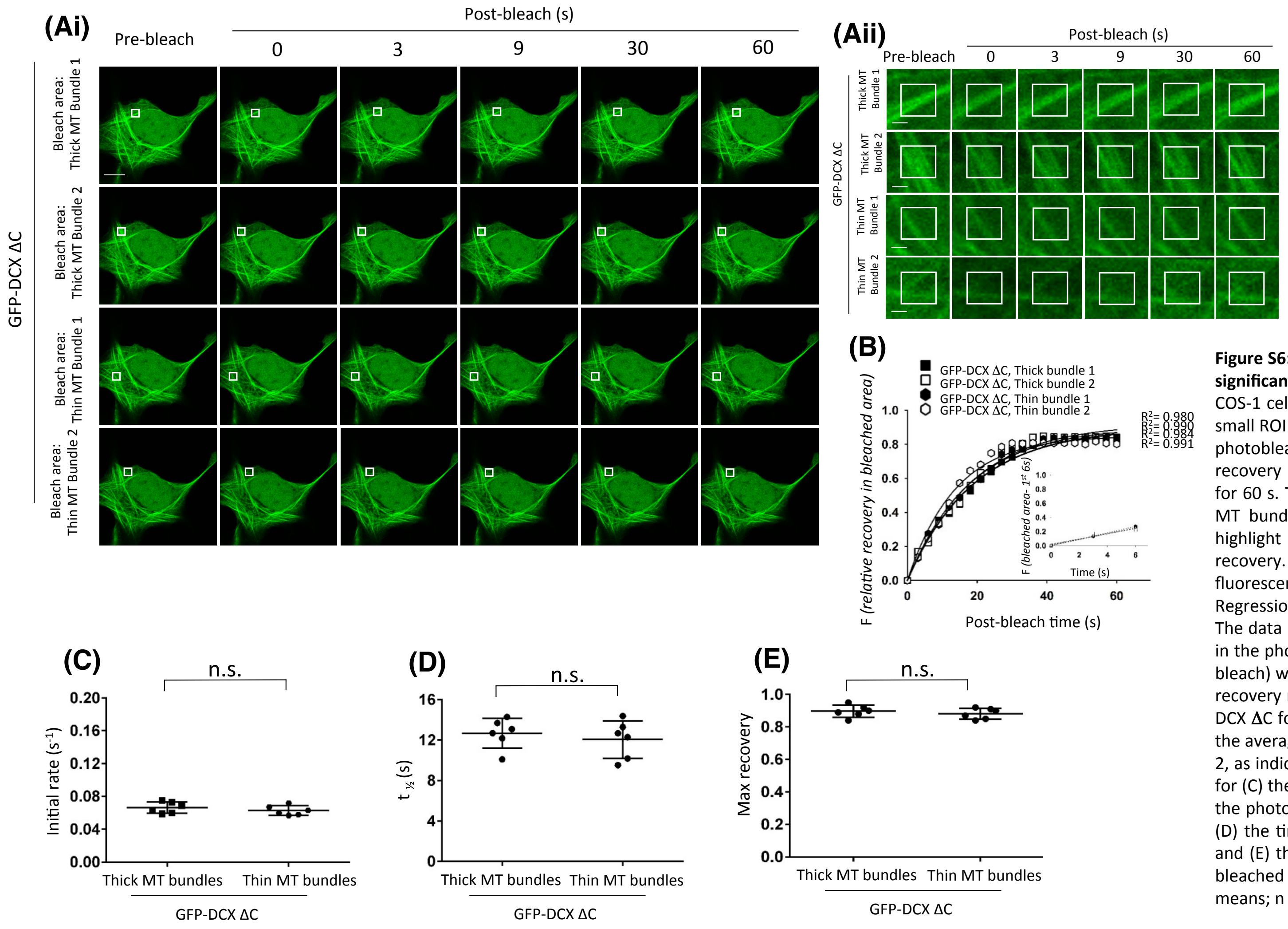

Figure S6: FRAP analysis shows that GFP-DCX ΔC dynamics are not significantly influenced by MT bundle thickness in individual cells. COS-1 cells were transfected to express GFP-DCX ΔC. (Ai) Different small ROI (indicated by the white rectangle in each cell image) were photobleached sequentially in the same cell and the fluorescence recovery was subsequently monitored post-bleach at 3s intervals for 60 s. Thin MT bundles were defined as < 1 μm width and thick MT bundles were defined as < 1 μm width. (Aii) Zoom images highlight the corresponding photobleached area fluorescence recovery. Scale bars represent 10 μm. (B) Plots of the recovery of fluorescence in the small area of bleach for GFP-DCX ΔC are shown. Regression values for the accuracy of each curve fit are indicated. The data in the inset represents the initial recovery of fluorescence in the photobleached area for first three time-points (0-6 s of post-bleach) with the line of best fit used for the calculation of the initial recovery rates. (C-E) The recovery of the ROI fluorescence for GFP-DCX ΔC for thick and thin bundles, where each data point shown is the average thickness of Thick Bundle 1 and 2, or Thin Bundle 1 and 2, as indicated in an individual cell. Results are for the mean ± SEM for (C) the initial rate of recovery of fluorescence for GFP-DCX ΔC in the photobleached area (estimated over the initial 6s post-bleach), (D) the time to reach half-maximal recovery of fluorescence ($t_{1/2}$), and (E) the fluorescence maximum recovery for GFP-DCXΔC in the bleached area. Error bars represent the standard error of the means; n = 6 cells. (n.s.= not significant).

Table S1

Primer	Sequence
XhoI-DCX FWD	5´-GCCTCGAGATGGAACTTGATTTTGGACACTTTGACGA-3´
XhoI-DCX REV	5´-GCCTCGAGTTACATGGAATCACCAAGCGA-3´
HindIII-DCX FWD	5´-GCAAGCTTATGGAACTTGATTTTGGACACTTTGACGA-3´
HindIII-DCX REV	5´-GCAAGCTTTTACATGGAATCACCAAGCGAG-3´
EcoRI-DCX FWD	5´-GCGAATTCATGGAACTTGATTTTGGACACTTTGACGA-3´
KpnI-DCX REV	5´-GCGGTACCTTACATGGAATCACCAAGCGAGTCCGAGTCATCCAA-3´
