# Supplementary material for: Dynamic microtubule association of Doublecortin X (DCX) is regulated by its C-terminus
Source: Sci Rep. 2017 Jul 12;7:5245. doi: 10.1038/s41598-017-05340-x (PMC5507856; doi:10.1038/s41598-017-05340-x)
Supplement: Supplementary file 2 — Key to Supplementary Movies S1-S4 [file 41598_2017_5340_MOESM2_ESM.pdf]

Dynamic microtubule association of Doublecortin X (DCX) is regulated by its C-terminus
Maryam Moslehi, Dominic C.H. Ng, and Marie A. Bogoyevitch
Key to Supplementary Movies

Movie S1

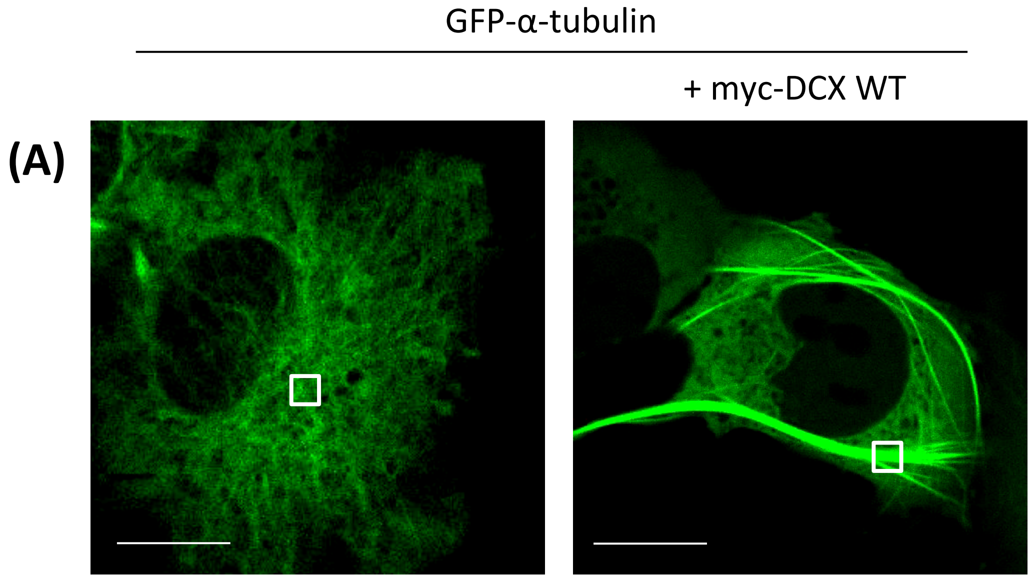

File: supp movie 1 A for GFP-tubulin.avi

File: supp movie 1 A for Myc-DCX WT and GFP-tubulin.avi

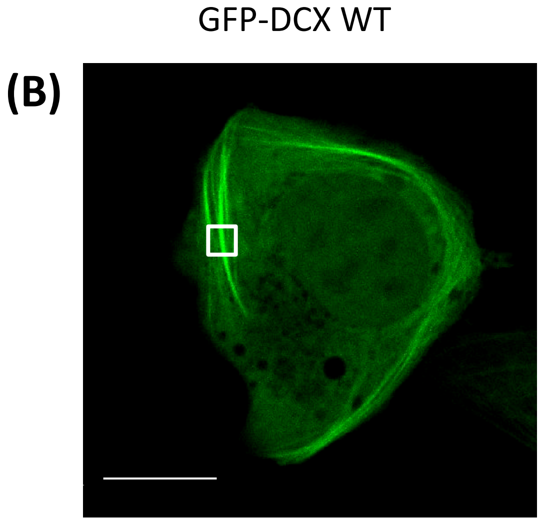

File: supp movie 1 B for GFP-DCX WT.avi

Movie S1: FRAP assessments of GFP-α-tubulin or GFP-DCX associations with the microtubule network. COS-1 cells were transfected to express (A) GFP-α-tubulin only or GFP-α-tubulin and myc-DCX WT to induce microtubule bundling and (B) GFP-DCX WT. S1A showed the fluorescence recovery of GFP-α-tubulin in the absence (left) and presence (right) of myc-DCX WT. S1B showed the fluorescence recovery of full-length GFP-DCX WT. In these movies, we monitored the fluorescence recovery of the mentioned constructs for 60 s (1 frame/s). Scale bars represent 10 μm. See Figure 1 for quantitative analyses.

Movie S2

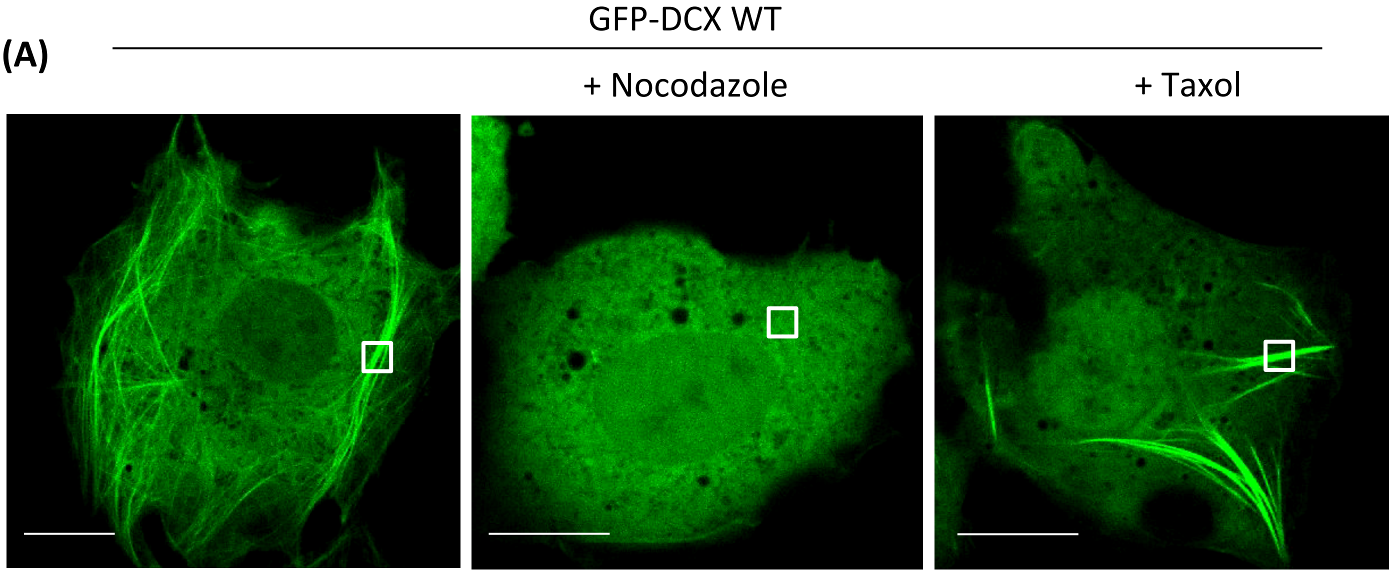

File:supp movie 2 A for GFP-DCX WT.avi

File: supp movie 2 A for Nocodazole-treated GFP-DCX WT.avi

File: supp movie 2 A for Taxol-treated GFP-DCX WT.avi

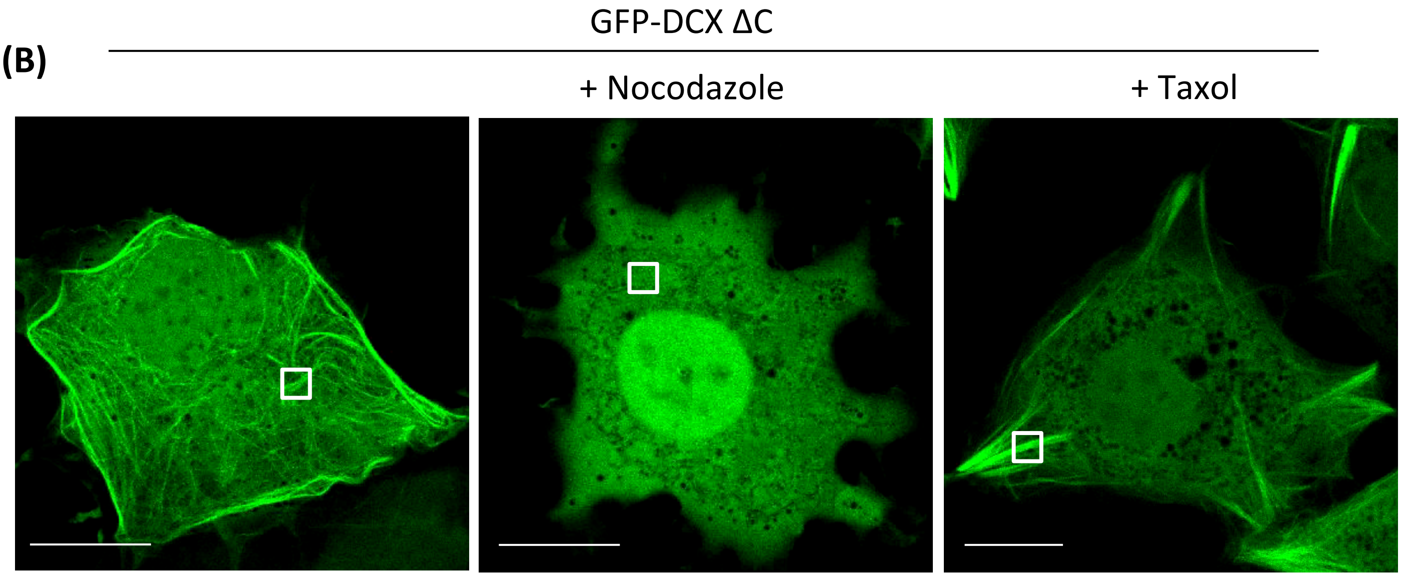

File: supp movie 2 B for GFP-DCX delta C.avi

File: supp movie 2 B for Nocodazole-treated GFP-DCX delta C.avi

File: supp movie 2 B for Taxol-treated GFP-DCX delta C.avi

Movie S2: Deletion of the DCX C-terminus slows DCX dynamics of association with microtubule bundles and decrease DCX's sensitivity to microtubule conformational changes. COS-1 cells were transfected to express (A) GFP-DCX WT and (B) GFP-DCX ΔC. (A) Fluorescence recovery was monitored for non-treated GFP-DCX WT (left), nocodazole-treated GFP-DCX WT (middle) and taxol-treated GFP-DCX WT (right) for 60 s. (B) Fluorescence recovery was monitored for non-treated GFP-DCX ΔC (left), nocodazole-treated GFP-DCX ΔC (middle) and taxol-treated GFP-DCX ΔC (right) for 60 s (1 frame/s). Scale bars represent 10 µm. See Figures 3 and 4 for quantitative analyses.

Movie S3

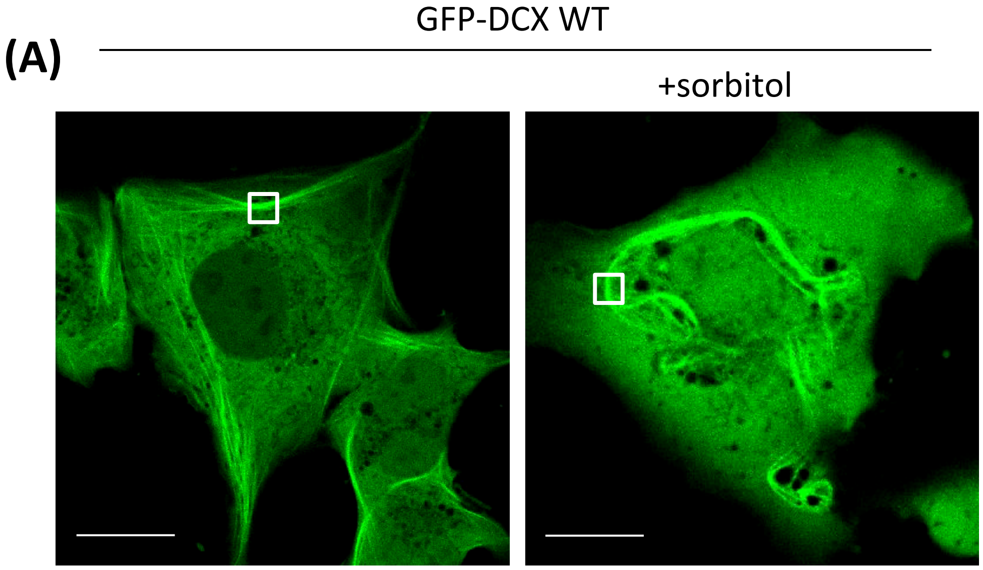

File: supp movie 3 A for GFP-DCX WT.avi

File: supp movie 3 A for Sorbitol-treated GFP-DCX WT.avi

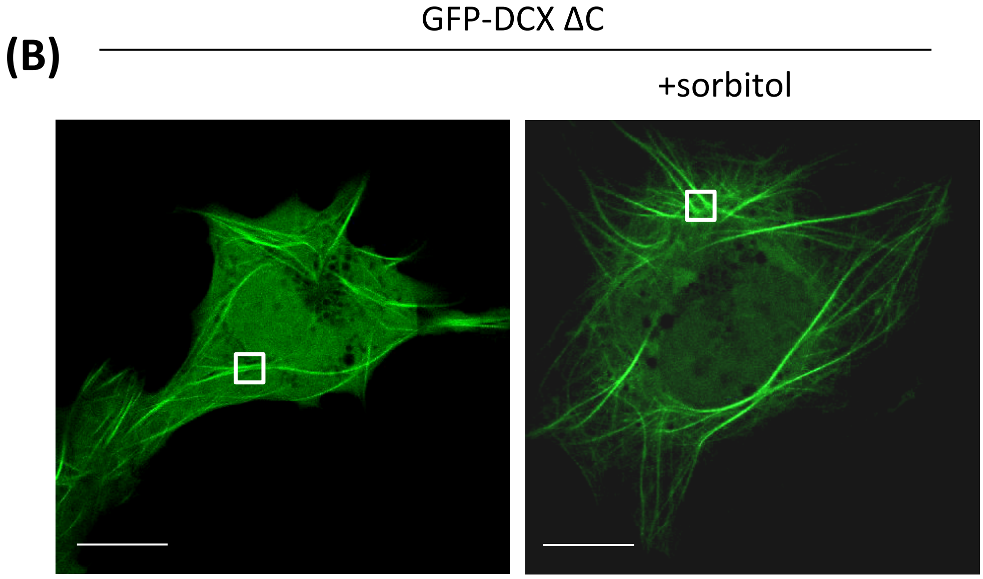

File: supp movie 3 B for GFP-DCX delta C.avi

File: supp movie 3 B for Sorbitol-treated GFP-DCX delta C.avi

Movie S3: Osmotic stress decreases DCX dynamic association with microtubules while DCX ΔC shows no difference in kinetics under basal and osmotic stress conditions. COS-1 cells were transfected to express (A) GFP-DCX WT and (B) GFP-DCX ΔC. Cells were treated with sorbitol (0.5M for 1 h) to induce hyperosmotic stress. (A) Fluorescence recovery was monitored for non-treated GFP-DCX WT (left) and sorbitol-treated GFP-DCX WT (right) for 60 s. (B) Fluorescence recovery was monitored for non-treated GFP-DCX ΔC (left) and sorbitol-treated GFP-DCX ΔC (right) for 60 s (1 frame/s). Scale bars represent 10 µm. See Figures 5 and 6 for quantitative analyses

Movie S4

(A) GFP-α-tubulin

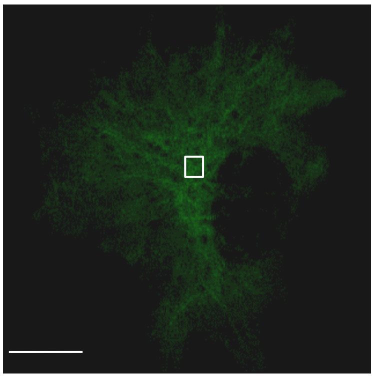

File: supp movie 4 A for GFP-tubulin.avi

(B) GFP-α-tubulin + sorbitol

	+ myc-DCX WT	+ myc-DCX ΔC
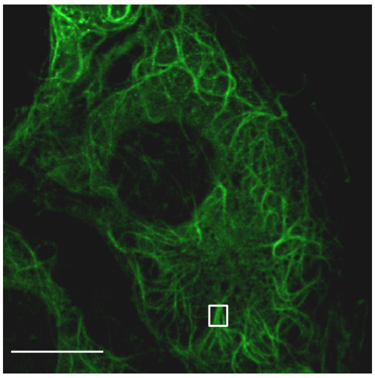	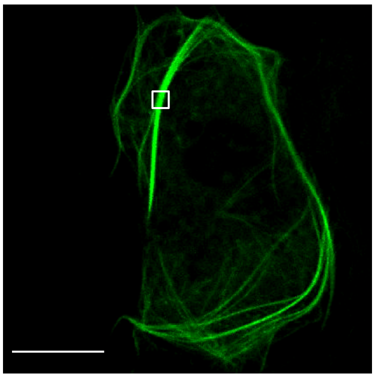	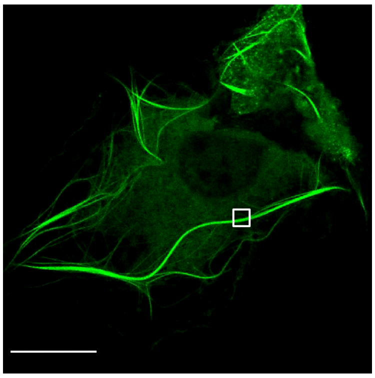
File: supp movie 4 B for Sorbitol-treated GFP-tubulin.avi	File: supp movie 4B for Sorbitol-treated Myc-DCX WT and GFP-tubulin.avi	File: supp movie 4B for Sorbitol-treated Myc-DCX deltaC and GFP-tubulin.avi

Movie S4: Osmotic stress conditions affect tubulin exchange dynamics. COS-1 cells were transfected to express GFP-α-tubulin, together with myc-DCX WT or myc-DCX ΔC as indicated. (B) Cells were treated with sorbitol (0.5M for 1 h) as indicated to induce hyperosmotic stress. Fluorescence recovery was monitored for 60 s (1 frame/s). Scale bars represent 10 µm. See Figures 7 and 8 for quantitative analyses
